# A Neural Circuit Mechanism for the Involvements of Dopamine in Effort-Related Choices: Decay of Learned Values, Secondary Effects of Depletion, and Calculation of Temporal Difference Error

**DOI:** 10.1523/ENEURO.0021-18.2018

**Published:** 2018-02-21

**Authors:** Kenji Morita, Ayaka Kato

**Affiliations:** 1 Physical and Health Education, Graduate School of Education, the University of Tokyo, Tokyo, 113-0033, Japan; 2 Department of Biological Sciences, Graduate School of Science, the University of Tokyo, Tokyo, 113-0033, Japan; 3 Department of Life Sciences, Graduate School of Arts and Sciences, the University of Tokyo, Tokyo, 153-8902, Japan; 4 Graduate Programme in Neuroscience, University of Oxford, South Parks Road, Oxford, OX1 3UD, United Kingdom

**Keywords:** Computational modeling, dopamine, effort, motivation, reinforcement learning, reward prediction error

## Abstract

Dopamine has been suggested to be crucially involved in effort-related choices. Key findings are that dopamine depletion (i) changed preference for a high-cost, large-reward option to a low-cost, small-reward option, (ii) but not when the large-reward option was also low-cost or the small-reward option gave no reward, (iii) while increasing the latency in all the cases but only transiently, and (iv) that antagonism of either dopamine D1 or D2 receptors also specifically impaired selection of the high-cost, large-reward option. The underlying neural circuit mechanisms remain unclear. Here we show that findings i–iii can be explained by the dopaminergic representation of temporal-difference reward-prediction error (TD-RPE), whose mechanisms have now become clarified, if (1) the synaptic strengths storing the values of actions mildly decay in time and (2) the obtained-reward-representing excitatory input to dopamine neurons increases after dopamine depletion. The former is potentially caused by background neural activity–induced weak synaptic plasticity, and the latter is assumed to occur through post-depletion increase of neural activity in the pedunculopontine nucleus, where neurons representing obtained reward exist and presumably send excitatory projections to dopamine neurons. We further show that finding iv, which is nontrivial given the suggested distinct functions of the D1 and D2 corticostriatal pathways, can also be explained if we additionally assume a proposed mechanism of TD-RPE calculation, in which the D1 and D2 pathways encode the values of actions with a temporal difference. These results suggest a possible circuit mechanism for the involvements of dopamine in effort-related choices and, simultaneously, provide implications for the mechanisms of TD-RPE calculation.

## Significance Statement

Depletion of dopamine (DA) or antagonism of either of the two major types of DA receptors was all shown to impair effortful actions to obtain large rewards while sparing reward-seeking or effort-exertion per se. DA has thus been proposed to play specific roles in reward-oriented effort exertion. However, underlying neural circuit mechanisms, and their relations with another popular role of DA, encoding of temporal-difference reward-prediction error (TD-RPE), remain unclear. We show that the experimental results suggesting DA’s involvements in effort-related choices can be consistently explained by the DA’s encoding of TD-RPE if assuming a mild decay of learned values, an increase of obtained-reward-representing input to DA neurons as a secondary effect of DA depletion, and a proposed circuit mechanism of TD-RPE calculation.

## Introduction

Dopamine (DA) has been suggested to be crucially involved in effort-related choices (Niv, 2007; Phillips et al., 2007; Salamone et al., 2007; Kurniawan et al., 2011). DA depletion was shown to change preference for a high-cost, large-reward option to a low-cost, small-reward option, but not when the large-reward option was also low-cost or the small-reward option gave no reward, while increasing the latency in all the cases, but only transiently (Salamone et al., 1994; Cousins et al., 1996). Antagonism of either dopamine D1 receptors (D1Rs; Nowend et al., 2001; Bardgett et al., 2009; Yohn et al., 2015) or D2 receptors (D2Rs; Salamone et al., 1994; Bardgett et al., 2009; Pardo et al., 2012) has also been shown to specifically impair the selection of high-cost, large-reward option. However, the underlying neural circuit mechanisms remain unclear. In particular, it is mysterious why the effects of DA depletion on choices were long lasting while those on the latency were transient. It is also nontrivial how DA depletion, D1R antagonism, and D2R antagonism all caused similar effects on choices, given the suggested distinct functions of the D1 and D2 corticostriatal pathways (Gerfen and Surmeier, 2011; Maia and Frank, 2011; Kravitz et al., 2012; Tai et al., 2012).

In parallel with studies examining roles of DA in effort-related choices, accumulated studies have suggested that DA represents TD-RPE (Schultz et al., 1997; Schultz, 2016), commonly across neurons (Eshel et al., 2016), with not only phasic but also tonic/sustained signals (Bromberg-Martin et al., 2010; Collins et al., 2016), and influencing learning behavior (Steinberg et al., 2013; Chang et al., 2016). Moreover, recent work has clarified the circuit mechanisms of RPE calculation (Cohen et al., 2012; Eshel et al., 2015; Keiflin and Janak, 2015) and DA/RPE-based learning (Yagishita et al., 2014). An emerging question is whether the effects of DA depletion and antagonisms in effort-related choices can be understood through DA’s role as TD-RPE, at least partially, and can in turn provide implications for mechanisms of TD-RPE calculation.

There have been attempts to explain the involvements of DA in effort-related choices in terms of reinforcement learning theory (Niv et al., 2007; Collins and Frank, 2014; Lloyd and Dayan, 2015). In particular, one study (Collins and Frank, 2014) considers that benefit and cost of an option are represented by the D1 and D2 basal-ganglia pathways, respectively, and DA depletion shifts the balance from the former to the latter, thereby causing a change in the preference from high-cost, large-benefit options to low-cost, small-benefit options. This model explained various experimental findings on both learning-related and motivational aspects of DA (Collins and Frank, 2014). Nonetheless, some of the experimental results—specifically, temporal changes in the latency, as well as in the choice ratio in a certain condition—remain to be explained. Also, this model does not consider the temporal difference–type RPE that has been suggested to be represented by the temporal change of DA signals within a trial as well as across trials (Montague et al., 1996; Schultz et al., 1997; Niv and Schoenbaum, 2008), and thus does not explain the temporal pattern of DA signals.

Under the assumption that DA represents TD-RPE and assuming that the learned action values mildly decay in time, we have recently shown that some of the results on the involvements of DA in effort-related choices, as well as the temporal pattern of DA signals, could be explained (Kato and Morita, 2016). However, temporal changes in the latency, and also choices in a certain condition, remained to be explained. Moreover, the effects of DA receptor antagonisms also remained to be explained because our previous model did not describe the D1 and D2 pathways. In the present work, we explored whether the results of DA depletion and antagonisms could be consistently explained by the DA’s representation of TD-RPE if possible secondary effects of DA depletion and proposed involvements of the D1 and D2 pathways in TD-RPE calculation were taken into account.

## Materials and Methods

### Code accessibility

We have uploaded the program codes to reproduce all the figure panels showing simulation results in this article, written in Matlab (MathWorks), in the ModelDB (https://senselab.med.yale.edu/modeldb/) with accession number 235045. The URL of the model is http://senselab.med.yale.edu/ModelDB/showModel.cshtml?model=235045, and the read-only access code is DpEf15704R17. The codes are also uploaded as Extended Data 1.

10.1523/ENEURO.0021-18.2018.ed1 Extended Data 1 Computer code: makefigures.m: running this file (script M-file) will reproduce all the figure panels showing simulation results in the article, as well as two supplementary figures (which are also attached here)
simTmaze.m: function M-file for simulating the effort-related T-maze task, which is called in makefigures.m
ana1.m–ana7.m: function M-files for analysis and plotting, which are called in makefigures.m
rantwi.mat: saved data (more specifically, outputs of rand(‘twister’)), which are used in makefigures.m to create exactly the same figures as presented in the article
mean2.m, std2.m, sem2.m: function M-files to calculate mean, std, and sem for input numbers omitting NaN
Fig. S1.pdf, Fig. S2.pdf: supplementary figures created by makefigures.m
How to run the makefigures.m files:
(1) Running makefigures.m will reproduce exactly the same results shown in the article (i.e., using the same sets of MATLAB-built-in pseudo-random numbers).
(2) Running makefigures.m with “samerand = 1” (line 4) changed to “samerand = 0” will result in conducting all the simulations with new sets of Matlab built-in pseudorandom numbers.
(3) Running makefigures.m with “mod_negativeTDE = 1” (line 19) changed to “mod_negativeTDE = 0” will result in conducting simulations assuming size reduction of TD-RPE–dependent value update by DA depletion with the assumption that the size reduction is applied only when TD-RPE is nonnegative. Download Extended Data 1, ZIP file.

### Simulation of the effort-related T-maze task

**Figure 1.**
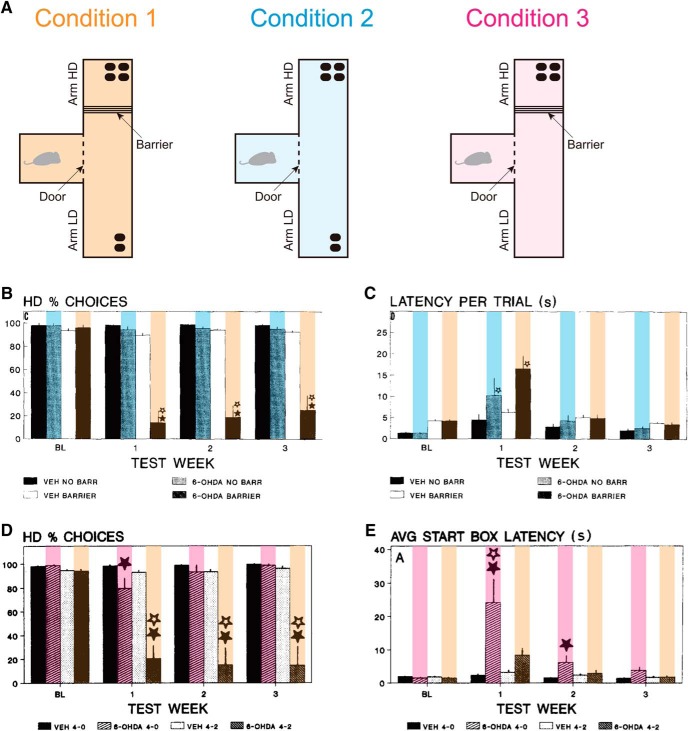
Experimental results on the effort-related T-maze choice task. Panels ***B***, ***C***, ***D***, and ***E*** were reprinted from Salamone et al. (1994), copyright 1994, and Cousins et al. (1996), copyright 1996, respectively, with permission from Elsevier; colors were added on the bars, also with permission. ***A***, Three task conditions. Condition 1: large and small reward were placed in the HD (high-reinforcement-density) and LD (low-reinforcement-density) arms, respectively, and a physical barrier was placed only in the HD arm. Condition 2: the same as Condition 1, except that there was no barrier in either arm. Condition 3: the same as Condition 1, except that the LD arm did not contain any reward. ***B***, The ratio of selecting the HD arm in Condition 1 (orange-marked bars) and Condition 2 (blue-marked bars) in Salamone et al. (1994). BL in the horizontal axis indicates the baseline period before dopamine (DA) depletion, and TEST WEEK 1, 2, and 3 indicate the first, second, and third week after injection of 6-OHDA that caused DA depletion. The bars without colors indicate the data for control animals injected with vehicle instead of 6-OHDA. ***C***, The latency of start-door opening in Condition 1 (orange-marked bars) and Condition 2 (blue-marked bars) in Salamone et al. (1994). ***D***, The ratio of selecting the HD arm in Condition 1 (orange-marked bars) and Condition 3 (pink-marked bars) in Cousins et al. (1996). ***E***, The latency of start-door opening in Condition 1 (orange-marked bars) and Condition 3 (pink-marked bars) in Cousins et al. (1996).

**Figure 2.**
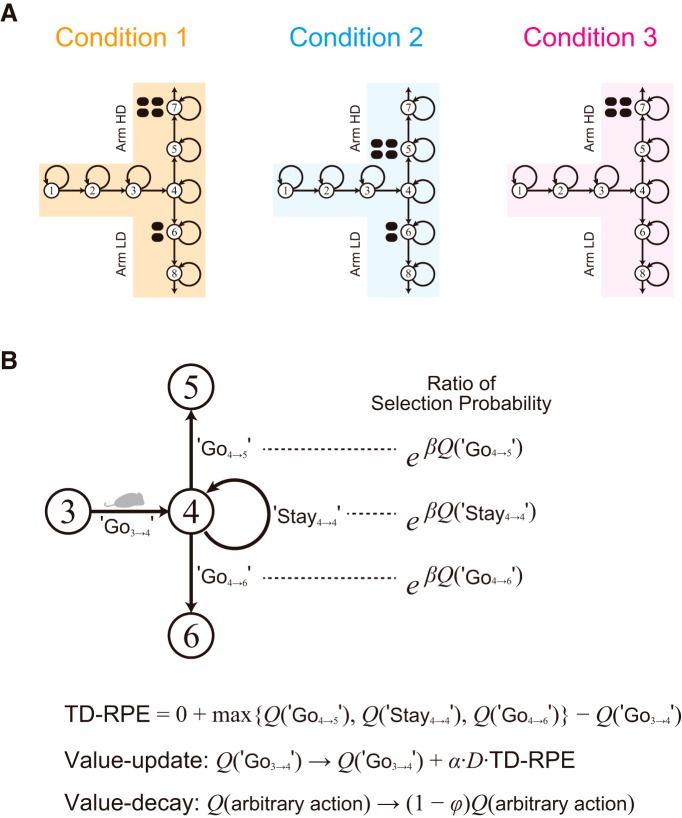
Simulation of the effort-related T-maze choice task by a reinforcement-learning model incorporating the decay of learned values. ***A***, Self-paced navigation in the T-maze was simulated by a series of selections of Go, move to the next state (indicated by the straight arrows), or Stay, stay at the same state (indicated by the round arrows). The physical barrier placed in the HD arm in Condition 1 and 3 in the experiments was represented as the existence of an extra state preceding the rewarded state in the HD arm, i.e., State 5 preceding State 7. ***B***, Magnification of the T-maze near the T-junction, illustrating a situation where the rat is taking Go from State 3 to State 4 (denoted as Go_3→4_). At the next time step, the rat arrives at State 4 and selects Go_4→5_ (go to the HD arm), Stay_4→4_, or Go_4→6_ (go to the LD arm) depending on the values of these actions, with the ratio of probabilities shown in the right. TD-RPE is calculated, and the value of Go_3→4_ is updated according to the TD-RPE, and in addition, the value of arbitrary action decays, as shown in the bottom. α, β, and φ in the formulas are the parameters representing the learning rate, inverse temperature (which determines the degree of exploitation over exploration on choice), and decay rate, respectively, and they were set to 0.5, 5, and 0.01 in the simulations. *D* in the formula of TD-RPE is the parameter for DA depletion: it was set to 1 before depletion (1–500 trials), and 0.25 after depletion (501–1000 trials).

We simulated the effort-related T-maze task (Fig. 1; see Results for explanation; Salamone et al., 1994; Cousins et al., 1996; Pardo et al., 2012; Yohn et al., 2015) by reinforcement learning (RL) models assuming the DA’s representation of TD-RPE. The T-maze was modeled as a set of states, each of which represented a particular location in the maze (Fig. 2*A*). At the beginning of each trial, the subject was assumed to be at State 1. Discrete time representation was assumed, and at each time step, the subject was assumed to select one of the possible actions according to its learned values in a soft-max manner (Daw et al., 2006). Specifically, action *A_i_* among possible actions (*i* = 1, …) was selected with probability *P*(*A_i_*) that was proportional to exp[β*Q*(*A_i_*)],

(1) $$P\left(A_{i}\right)=\frac{e^{\beta Q\left(A_{i}\right)}}{\sum_{j}e^{\beta Q\left(A_{j}\right)}},$$

where *Q*(*A_i_*) was the learned value of action *A_i_*, and β was a parameter called the inverse temperature representing the degree of exploitation over exploration on choice and was set to 5 in all the simulations. At each state except for the state at the T-junction (State 4) and the end-state (not illustrated in the figure: see below), there were two possible actions: Go (move to the next state) and Stay (stay at the same state). This Go or Stay (or No-Go) selection described the self-paced nature of the task (Kato and Morita, 2016). At State 4, there were three possible actions (Fig. 2*B*): Choose the HD (high-reinforcement-density) arm and Go to State 5 (referred to as Go_4→5_), choose the LD (low-reinforcement-density) arm and Go to State 6 (referred to as Go_4→6_), and Stay (stay at State 4). When subject took Go at State 7 or 8, subject was assumed to move to the end-state (not illustrated in the figure), and then move back to State 1 at the next time step, and the next trial started.

In Condition 1 (Fig. 2*A*, left), large reward (size 1) was assumed to be given when subject reached State 7 for the first time in a trial, whereas small reward (size 0.5) was given when reaching State 6 for the first time in a trial. The physical barrier placed in the HD arm in the experiment was represented as the existence of an extra state preceding the rewarded state in the HD arm, i.e., State 5. In Condition 2, there was no barrier in the experiment, and so large reward was assumed to be given at State 5 in the HD arm in the model (Fig. 2*A*, middle). In Condition 3 (Fig. 2*A*, right), large reward was given at State 7 in the HD arm and no reward was given in the LD arm, as in the experiment. In addition to these three conditions that were originally examined (Salamone et al., 1994; Cousins et al., 1996), we also simulated another condition examined in a recent study (Pardo et al., 2012; Condition 4: Fig. 10*A*, right), in which a physical barrier was placed in both the HD and LD arms, and in the model, large reward was given at State 7 and small reward was given at State 8 (Fig. 12*D, a*).

At every time step, TD-RPE (TD error; Sutton and Barto, 1998) was calculated as

(2) $$TD\text{-}RPE\left(t\right)=R\left(t\right)+Q_{\text{Upcoming}}-Q_{\text{Previous}},$$

where *R*(*t*) was the obtained reward, which was 0 unless the subject reached a rewarded state for the first time in a trial, and *Q_Upcoming_* and *Q_Previous_* were the upcoming and previous values, respectively, was calculated according to an RL algorithm called the Q-leaning (Watkins, 1989):

(3) $$TD\text{-}RPE\left(t\right)=R\left(t\right)+\max\left[Q\left(A_{i}\right)\right]-Q\left[A\left(t-1\right)\right],$$

where *A_i_* (*i* = 1, …) were possible upcoming actions and *A*(*t* – 1) was the action taken at the previous time step, except at State 1 where the previous action was not defined and the *Q*[*A*(*t* – 1)] term in the above equation was replaced with 0. The learned value of the previous action was assumed to be updated according to the TD-RPE (except at State 1):

(4) Q[A(t−1)]→Q[A(t−1)]+αTD-RPE(t),

where α was a parameter representing the learning rate and was set to 0.5 in all the simulations. In addition, the learned value of every action was assumed to decay at a constant rate at every time step:

(5) Q(A)→(1−φ)Q(A),

where φ was a parameter representing the decay rate and was set to 0.01 (i.e., 1% of the current value) in all the simulations shown in the figures on this manuscript; φ was set to 0.001 in separate simulations, whose results can be seen in the ModelDB (Fig. S1). Such a decay (forgetting) of learned values was shown to explain the experimentally observed ramping pattern of DA signals (Morita and Kato, 2014) and motivational functions of DA (Kato and Morita, 2016). Notably, temporal discounting was not assumed: see Kato and Morita (2016) for discussion on how the decay of learned values could be regarded as a partial implementation of temporal discounting. We will discuss possible rationale and mechanisms for the decay in the Discussion.

For each of condition of the task (see Results), the learned values of all the actions were initially set to 0, and 500 trials were simulated. Subsequently, DA depletion, without or with possible secondary effects, or D1R or D2R antagonism was incorporated (see below), and another 500 or 1500 trials were simulated. For each combination of task condition and assumption about depletion, secondary effects, or antagonism, simulation of in total 1000 or 2000 trials was executed 20 times with different sets of pseudorandom numbers. In the simulations for Fig. 8*C* and Fig. 9, action values became extremely large in some cases, and therefore in all the simulations shown in these figures, simulation was quitted when action value larger than 100 times of the size of the large reward was detected. Simulations were performed using Matlab (MathWorks). Standard errors shown in the figures were calculated by dividing the standard deviations by the square root of the number of simulation runs that were completed and included.

### Incorporation of DA depletion

**Figure 3.**
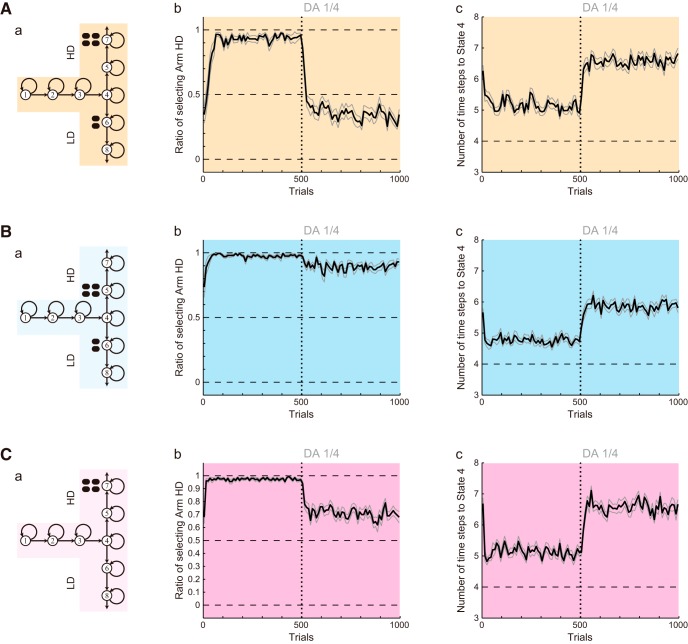
Results of the simulations of the effort-related T-maze choice task by the model considered in our previous study (Kato and Morita, 2016). ***A***, ***B***, and ***C*** show the results of Conditions 1, 2, and 3, respectively. The panels of ***A*** and ***B*** were reused from Kato and Morita (2016) under license. ***a***, Schematics of the simulated task conditions. ***b***, The ratio of choosing the HD arm in each successive 10 trials. The black thick line and the gray thin lines indicate the mean ± SEM of 20 simulations, and the vertical dotted line indicates the onset of DA depletion (the same notations are also applied to ***c***). ***c***, The latency (number of time steps) of reaching the T-junction (State 4), averaged over each successive 10 trials.

Given the assumption that DA represents TD-RPE, we first incorporated DA depletion into the model as a reduction of the size (i.e., absolute value) of TD-RPE–dependent update of learned values. In the T-maze experiment (Salamone et al., 1994), neurochemical analyses revealed that the DA content in the nucleus accumbens (NAc) in the rats injected with 6-hydroxydopamine (6-OHDA) was reduced to 20.3∼23.7% of the content in the control rats injected with ascorbate vehicle (this analysis was conducted after the T-maze experiment: see Results for discussion related to this point). In our model, DA depletion was assumed to cause a reduction of the size of TD-RPE–dependent update of learned values to 25% of the original size (i) only when TD-RPE was nonnegative, and in separate sets of simulations, (ii) regardless of whether TD-RPE was nonnegative or negative. In the simulations shown in Figs. 3, 5, 6, 7, 8*A–C*, *Dd–g*, and S1, and the gray lines in Fig. 15, TD-RPE was always nonnegative and thus results for i and those for ii should be the same; practically, results for (i) were used to plot Fig. 3, 5, 6, 7, 8*A–C*, *Dd–g* , whereas results for ii were used to plot Fig. S1 and the gray lines in Fig. 15. In the simulations shown in Fig. 8*Db*,*c* and *9* , results for ii are shown (results for i can be obtained by using the codes uploaded in the ModelDB).

Although we originally modeled DA depletion in the above manner, it would be possible that DA depletion instead or in addition causes modulations of the responsiveness of striatal neurons and DA axons expressing D1Rs/D2Rs. Therefore we also examined this possibility by performing separate sets of simulations assuming the same effects as assumed for D1R and D2R antagonisms described below (Fig. 14*B*) or these effects plus the reduction of the size of TD-RPE–dependent value update to 25% (Fig. 14*C*) or 50% (Fig. 14*D* and the purple-gray lines in Fig. 15) of the original size regardless of whether TD-RPE was nonnegative or negative.

### Incorporation of possible secondary effects of DA depletion

There is ample evidence that DA depletion causes secondary, potentially compensatory, effects (Bezard et al., 2003; Rivlin-Etzion et al., 2006). One of the secondary effects observed in rats injected with 6-OHDA is the increase in the firing rate of neurons in the pedunculopontine nucleus (PPN; Breit et al., 2001; Zhang et al., 2008; Geng et al., 2016), which sends excitatory [glutamatergic (Yoo et al., 2017) and cholinergic (Dautan et al., 2016; Xiao et al., 2016)] projections to DA neurons, although some studies reported no change (Aravamuthan et al., 2008) or a decrease (Florio et al., 2007) of the PPN firing rate. In the studies showing the increase of the PPN firing rate (Breit et al., 2001; Zhang et al., 2008; Geng et al., 2016), recording was made ∼3 wk after 6-OHDA injection and the increase was observed. Given that changes in the firing rate would occur gradually rather than abruptly, it would be reasonable to assume that increase began before ∼3 wk. This is a time scale matching the duration of the T-maze experiments that we simulated (Salamone et al., 1994; Cousins et al., 1996). Although the location of 6-OHDA injection differed between studies showing the increase of the PPN firing rate [the substantia nigra pars compacta (SNc; Breit et al., 2001; Zhang et al., 2008) or the medial forebrain bundle (MFB; Geng et al., 2016)] and the T-maze experiments (NAc), we assumed that similar increases of the PPN firing rate occurred in the T-maze experiments, and through them, the gain of the excitatory input from PPN to DA neurons increased (see Discussion for more on this assumption).

PPN has been shown to contain two types of reward-related neurons (Okada et al., 2009): type 1 showing sustained activity between cue and reward with the level scaling with the predicted reward size, and type 2 showing phasic activity after reward delivery with the level scaling with the actual reward size. type 2 has been proposed (Kawato and Samejima, 2007; Okada et al., 2009; Morita et al., 2012) to send information about the obtained reward to the DA neurons via excitatory projections, providing the obtained-reward term [*R*(*t*)] of TD-RPE. Some hypotheses proposed that type 1 also contributes to the TD-RPE calculation by providing the previous-value term (–*Q_Previous_*; Cohen et al., 2012), upcoming-value term (*Q_Upcoming_*), or both (Kawato and Samejima, 2007; Okada et al., 2009), while others (e.g., Morita et al., 2012) proposed that the previous and upcoming values come from other sources. Considering these, we assumed that the gain of one or more terms of TD-RPE gradually increased after DA depletion. We first tested four cases with the gain increase of (1) the obtained-reward term only, (2) all three terms, (3) the obtained-reward and upcoming-value terms, and (4) the obtained-reward and previous-value terms. We next assumed that the gains of the inputs representing the obtained reward, upcoming value, and previous value increased up to *x*, *y*, and *z* times, respectively, and simulations were conducted with the parameters *x*, *y*, and *z* were systematically varied (1, 1.5, 2, 2.5, or 3).

**Figure 4.**
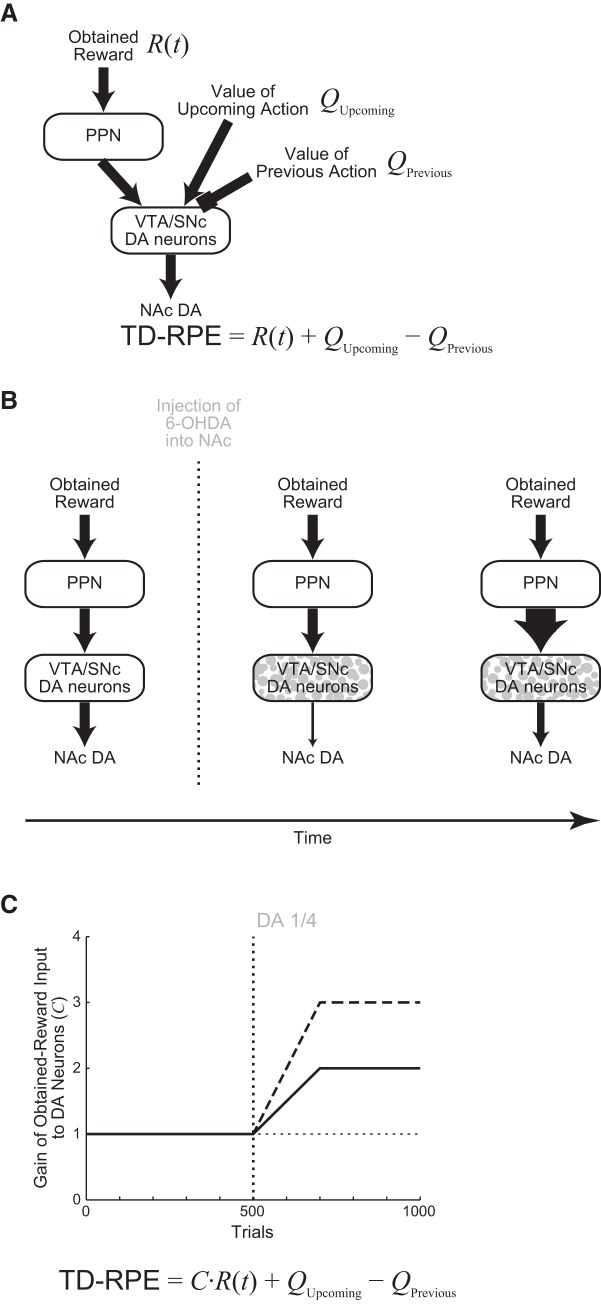
Incorporation of possible secondary effects of DA depletion into the model. ***A***, A circuit mechanism of TD-RPE calculation in DA neurons that has been suggested, in which DA neurons receive input representing the obtained reward from a subpopulation of the PPN neurons. VTA, ventral tegmental area; SNc, substantia nigra pars compacta; NAc, nucleus accumbens. ***B***, Schematic diagram of the secondary effect that we incorporated into the model. The PPN neuronal activity representing the obtained reward was assumed to gradually increase after DA depletion. ***C***, Assumed gradual increase in the gain of the obtained-reward-representing input to DA neurons, corresponding to the coefficient (*C*) of the obtained-reward term in the formula of TD-RPE as shown in the bottom. The solid and dashed lines indicate the gain increase up to twice and three times of the original assumed in the simulations in Figs. 5 and 6 and Fig. 8, respectively.

We assumed that the gradual gain increase lasted for 200 trials and then reached a plateau (Fig. 4*C*), considering that (1) in the T-maze experiments (Salamone et al., 1994; Cousins et al., 1996), rats executed the task 30 trials/d and 5 d/wk, and behavior over 3 wk after 6-OHDA injection was recorded, and (2) in the studies reporting the increase of PPN neuronal activity after DA depletion (Breit et al., 2001; Zhang et al., 2008; Geng et al., 2016), the recordings were made ∼3 wk after 6-OHDA injection. The level of the plateau was first set to twice of the original, considering that the reported increase of the mean firing rate of PPN neurons after DA depletion was ∼1.7-fold (Breit et al., 2001), ∼1.1- and ∼1.8-fold (presumed cholinergic and noncholinergic neurons, respectively; Zhang et al., 2008), or 1.3∼1.8-fold depending on the neuron type and the animal’s state (Geng et al., 2016). Subsequently, we assumed that the level of the plateau was 1∼4 times of the original, which is also considered to be plausible given that neuronal input-output transformation is nonlinear and that DA depletion was reported to cause changes in the PPN firing pattern, in addition to the firing rate (Breit et al., 2001; Zhang et al., 2008; Geng et al., 2016).

### Incorporation of D2R or D1R antagonism

Activation of D2Rs on the DA axons inhibits DA release, causing a negative feedback, and D2R antagonist relieves such an inhibition (Gonon and Buda, 1985) and also inhibits DA uptake (Benoit-Marand et al., 2011), causing an enhancement of DA signaling. Because DA is assumed to represent TD-RPE in our model, we incorporated the D2R antagonist-induced enhancement of DA signaling into the model as 1.25-times amplification of TD-RPE–dependent update of action values.

D2Rs are also expressed in about half of the striatal medium spiny neurons (MSNs), while the other half of MSNs express D1Rs (Gerfen and Surmeier, 2011). Activation of D2Rs causes a reduction of the responsiveness of D2-MSNs (Gerfen and Surmeier, 2011), and D2R antagonist is considered to block such a reduction and thereby amplify the output of D2-MSNs. Recent work (Pardo et al., 2012) has shown that the behavioral effect of D2R antagonism in the T-maze experiment was attenuated by administration of the antagonist of adenosine A2A receptors (A2ARs), and also that striatal c-Fos induction by D2R antagonism was attenuated by A2AR antagonism. A2ARs are selectively expressed in D2-MSNs (Fink et al., 1992), and antagonism of A2ARs has been shown to impair the long-term potentiation of excitatory synapses on D2-MSNs (Shen et al., 2008). Therefore, the attenuation of the effect of D2R antagonism by A2AR antagonism (Pardo et al., 2012) suggests a crucial involvement of D2Rs on D2-MSNs in effort-related choices in the T-maze experiment. Regarding possible relations between D2-MSNs and TD-RPE, the cortico-striatal temporal difference (CS-TD) hypothesis (Morita et al., 2012, 2013; Morita, 2014; Morita and Kawaguchi, 2015) posits that D2-MSNs represent the value of previous action (or state) and negatively impact the DA neurons via the indirect pathway of the basal ganglia, providing the previous-value term (−*Q_Previous_*) of TD-RPE. Although there are controversial issues regarding the selectivity of corticostriatal connections (Kress et al., 2013; Morita, 2014; Shipp, 2017) and plasticity of corticostriatal synapses (Morita et al., 2013; Morita and Kawaguchi, 2015), we assumed this CS-TD hypothesis. Accordingly, we assumed that D2R antagonist caused 1.25-times amplification of the previous-value term of TD-RPE, in addition to the amplification of the TD-RPE–dependent value update described above.

As mentioned above, D1Rs are expressed in the other half of MSNs, and activation of D1Rs causes an enhancement of the responsiveness of D1-MSNs (Gerfen and Surmeier, 2011). D1R antagonist is considered to block such an enhancement and thereby reduce the output of D1-MSNs. The CS-TD hypothesis, introduced above, posits that D1-MSNs represent the value of upcoming action (or state) and positively impact the DA neurons, providing the upcoming-value term (*Q_Upcoming_*) of TD-RPE. According to this, we assumed that D1R antagonist caused a reduction of the upcoming-value term of TD-RPE to 0.8 times the original. Notably, in any case, we assumed that D1R or D2R antagonist at the doses used in the studies that we simulated [0.1 mg/kg haloperidol (D2R antagonist) in Salamone et al. (1994) and 0.1–0.3 mg/kg ecopipam (D1R antagonist) in Yohn et al. (2015)] changed the activity of MSNs and/or DA axons and thereby changed TD-RPE as described above but did not directly affect the induction of DA-dependent plasticity. Also, effects of the antagonists on D1Rs or D2Rs in other regions including the cerebral cortex were not considered in the model.

### Incorporation of modulations of MSNs’ responsiveness by DA/TD-RPE at the previous time step

In the above, we described how modulations of the responsiveness of MSNs by DA antagonisms and/or depletion were incorporated into the model as changes in the calculation of TD-RPE, while we did not assume that the responsiveness of MSNs is in turn modulated by TD-RPE that is assumed to be represented by DA. Although there could be a distinction such that DA antagonisms or depletion regards tonic DA whereas TD-RPE regards phasic DA, our model does not distinguish tonic DA and phasic DA, and TD-RPE–representing DA could still modulate the responsiveness of MSNs. To examine this possibility, we performed separate sets of simulations of the DA depletion experiments in which the responsiveness of D1 MSNs and D2 MSNs was assumed to be affected by TD-RPE at the previous time step. More specifically, in those simulations [data not shown; the codes and resulting figure (Fig. S2) can be seen in the ModelDB], the upcoming-value and previous-value terms of TD-RPE(*t*) were multiplied by *c*_1_ × [1 + 0.1 × *c*_0_ × TD-RPE(*t* − 1)] and *c*_2_ × [1 − 0.1 × *c*_0_ × TD-RPE(*t* − 1)], respectively, where *c*_0_, *c*_1_, and *c*_2_ were set as follows. In the cases without DA depletion, *c*_0_ = *c*_1_ = *c*_2_ = 1. In the cases with DA depletion modeled as a reduction of the size of TD-RPE–dependent value update to 0.25 times, *c*_0_ = 0.25 and *c*_1_ = *c*_2_ = 1. In the cases with DA depletion modeled as a reduction of the size of TD-RPE–dependent value update to 0.5 times and modulations of MSNs/DA axons, *c*_0_ = 0.5 × 1.25, *c*_1_ = 0.8, and *c*_2_ = 1.25.

### Simulation of a different cost–benefit decision-making task

By using the same model as above (without DA depletion, secondary effects, or antagonism), we qualitatively simulated a different cost–benefit decision-making task examined in Gan et al. (2010), in which rats were trained to make either a benefit (reward)-based choice, choosing a cue associated with larger or smaller benefit (number of food pellets) with the cost (number of lever presses) equalized, or a cost (effort)-based choice, choosing a cue associated with smaller or larger cost with the benefit equalized. Specifically, we assumed reward 0.5 and 0.25 on States 6 and 5, respectively, to simulate benefit-based choices (Fig. 16*Ca*), or reward 0.25 on States 6 and 7, respectively, to simulate cost-based choices (Fig. 16*Cb*). We also simulated forced trials in the experiments, where only one of the two options in the choice trials was available, by disabling Go action from the T-junction to an arm corresponding to unavailable option, i.e., Go_4→5_ for forced trials with larger benefit (Fig. 16*Da*) or smaller cost (Fig. 16*Dc*) or Go_4→6_ for forced trials with smaller benefit (Fig. 16*Db*) or larger cost (Fig. 16*Dd*). Notably, whereas choice trials and forced trials were intermingled in the experiments, or more specifically, blocks of four forced trials and subsequent four choice trials were repeated in sessions where DA recording was made in the experiments (Gan et al., 2010), we simulated different types of forced trials separately from each other and also separately from choice trials. Also notably, in the model, the larger-benefit forced trials (Fig. 16*Da*) were identical to the smaller-cost forced trials (Fig. 16*Dc*), and thus we conducted only a single set of simulations that corresponded to both types of forced trials. The same set of parameters (learning rate α = 0.5, inverse temperature β = 5, value-decay φ = 0.01, and no temporal discounting) used for the simulations of the T-maze task were used, and 1000 trials were simulated for 20 times for each condition.

## Results

### Simulation of the DA depletion experiments and motivation for considering the secondary effects

A representative experimental paradigm to test roles of DA in effort-related choices is the T-maze task (Salamone et al., 1994; Cousins et al., 1996), which consisted of three conditions (Fig. 1*A*). In Condition 1, large reward was placed in one arm (HD, high-reinforcement-density arm), whereas small reward was placed in the other arm (LD, low-reinforcement-density arm), and a physical barrier was placed only in the HD arm. Intact rats preferred the HD (i.e., high-cost, large-reward) arm. However, after DA was depleted by intra-accumbens injection of 6-hydroxydopamine (6-OHDA), the rats changed their preferences to the LD (i.e., low-cost, small-reward) arm (Fig. 1*B*, orange-backed bars). In Condition 2, in which there was no barrier in the HD arm, DA depletion hardly changed the preference for the HD arm (Fig. 1*B*, blue-backed bars). In Condition 3, where the LD arm did not contain any reward, DA depletion mildly weakened the preference for the HD arm, but only transiently (Fig. 1*D*, pink-backed bars). In all three conditions, whether in the presence or absence of a preference change, DA depletion increased the latency of start-door opening (Fig. 1*C*,*E*), although this effect was also transient. These results, together with the results in a different paradigm (Salamone et al., 1991; Cousins and Salamone, 1994), have been interpreted that DA specifically serves for reward-oriented effort exertion rather than reward evaluation or effort exertion per se (Salamone et al., 2007).

First, we simulated the effort-related T-maze choice task (Salamone et al., 1994; Cousins et al., 1996) by the model considered in our previous study (Kato and Morita, 2016). The model describes this task as a set of states, each of which represents a particular location in the maze (Fig. 2*A*). The simulated subject selects Go action to go to a next state or Stay action to stay at the same state depending on the learned values of actions. The value of taken action is updated according to TD-RPE, while all the learned values are subject to a mild decay (Fig. 2*B*). DA depletion was assumed to cause a reduction of the size of nonnegative TD-RPE–dependent value increment to a quarter of the original size (TD-RPE was always nonnegative in the simulations shown in Fig. 3). Fig. 3 shows the simulation results on the ratio of choosing the HD arm (panels *b*) and the latency (number of time steps) of reaching the T-junction (State 4; panels *c*) in the three conditions [the results for Condisions 1 and 2 were already reported in Kato and Morita (2016)]. In Condition 1, DA depletion drastically changed the preference for the HD arm to the LD arm (Fig. 3*Ab*). By contrast, in Condition 2, DA depletion did not largely change the preference (Fig. 3*Bb*). These results are consistent with the experimental results (Fig. 1*B*), as shown in our previous study (Kato and Morita, 2016). In Condition 3, however, although DA depletion caused only a weak and transient reduction in the preference for the HD arm in the experiment (Fig. 1*D*), a more prominent and persistent decrease was caused in the simulation (Fig. 3*Cb*). Moreover, regarding the latency, although DA depletion caused only a transient increase in all the three conditions in the experiments (Fig. 1*C*,*E*), a persistent increase was caused in the simulations [Fig. 3, panels *c*; as previously shown for Conditions 1 and 2 (Kato and Morita, 2016)]. In this way, the previously considered model could reproduce some results, but not others, of the experiments.

**Figure 5.**
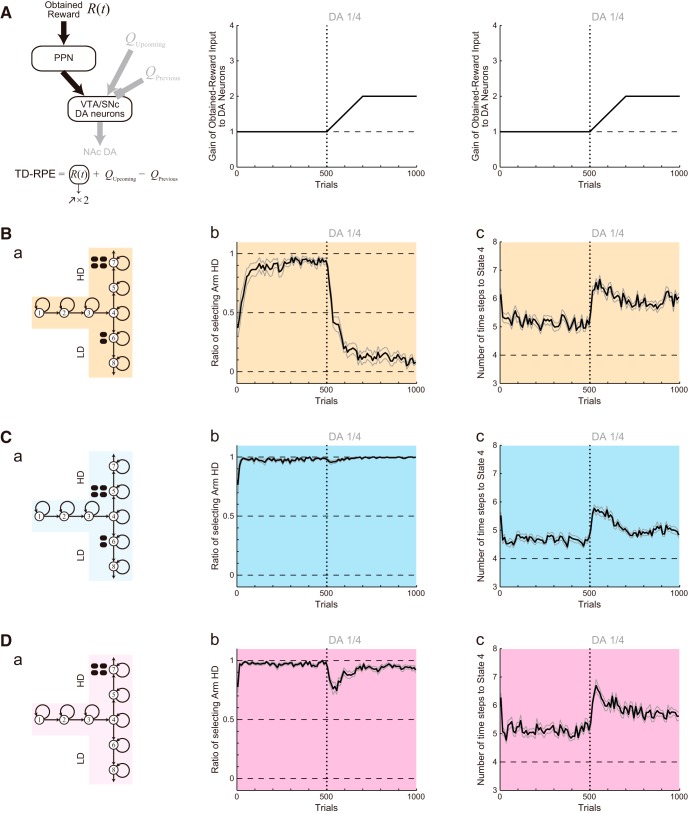
Results of the simulations of the effort-related T-maze choice task with the assumption that the gain of the obtained-reward-representing input to DA neurons gradually increases up to twice of the original after DA depletion. ***A***, Assumed gradual increase in the gain of the obtained-reward-representing input to DA neurons (the middle and the right graphs are identical to one another). **B–D**, Simulation results for Conditions 1, 2, and 3. The configurations are the same as those in Fig. 3. ***a***, Schematics of the simulated task conditions. ***b***, The ratio of choosing the HD arm. ***c***, The latency (number of time steps) of reaching the T-junction (State 4).

**Figure 6.**
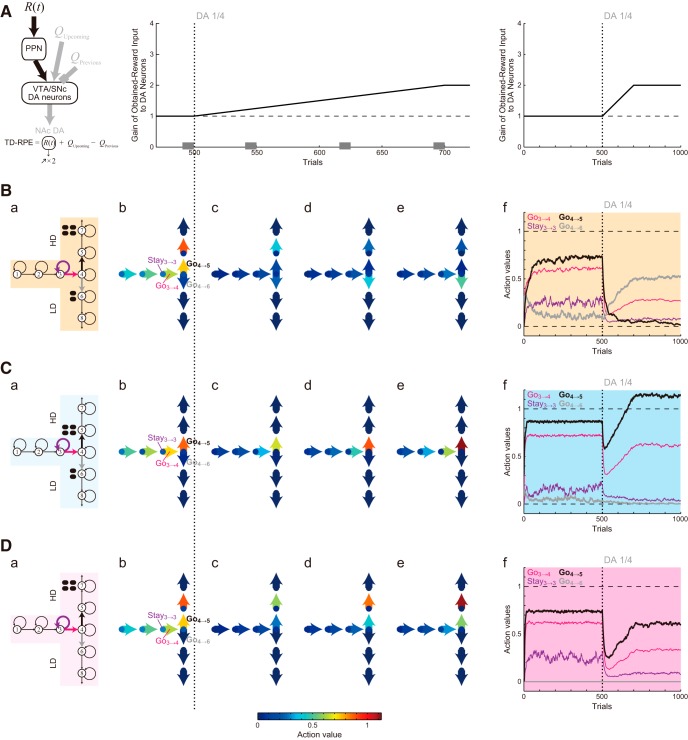
Evolutions of the learned values of actions in the simulations shown in Fig. 5. ***A***, Assumed gradual increase in the gain of the obtained-reward-representing input to DA neurons (the middle graph is a magnification of the right graph). ***B–D***, Results for Conditions 1, 2, and 3. ***a***, Schematics of the simulated task conditions. ***b–e***, The color arrows and circles along the T-shape indicate the learned values of Go actions and Stay actions, respectively, for the last 10 trials of 500 (***b***), 550 (***c***), 625 (***d***), and 700 (***e***) trials, averaged across 20 simulations, in reference to the bottom color scale bar. ***f***, Trial-by-trial evolutions of the learned values of Go_3→4_ (pink line), Stay_3→3_ (purple line), Go_4→5_ (thick black line), and Go_4→6_ (thick gray line) averaged across 20 simulations.

**Figure 7.**
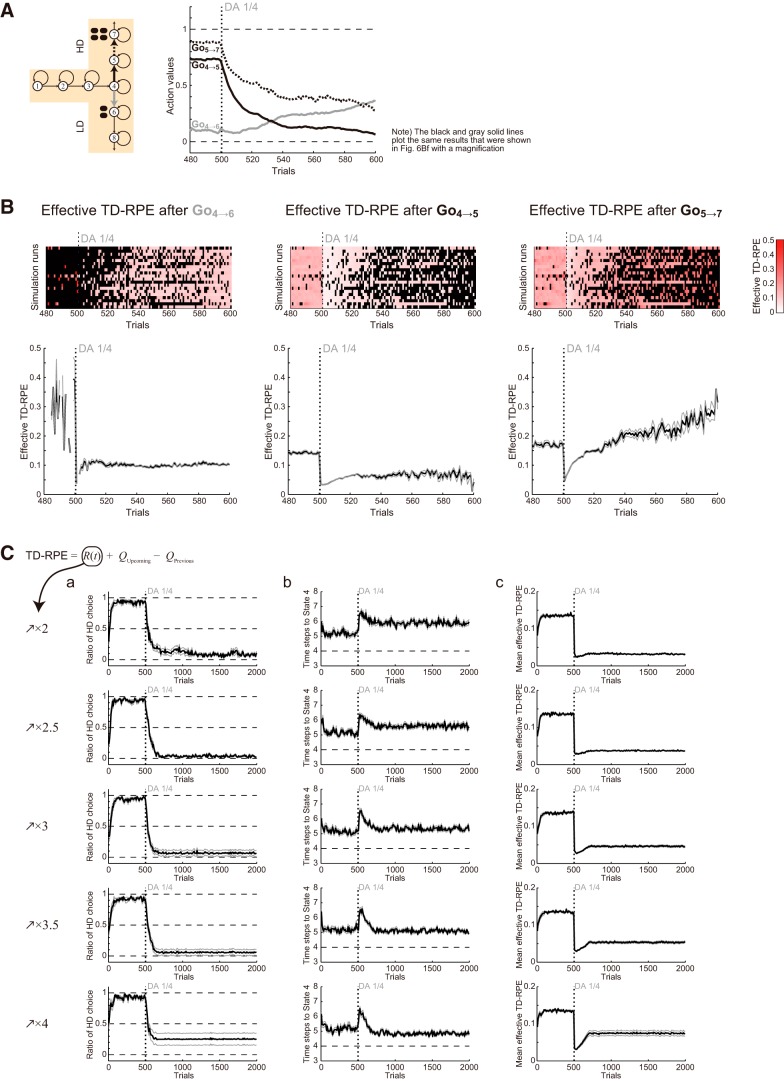
Detailed evolutions of the action values, the effective TD-RPEs, the ratio of choosing the HD arm, and the latency of reaching the T-junction after DA depletion in Condition 1. ***A***, Left: Schematics of the simulated task condition. Right: Trial-by-trial evolutions of the learned values of Go_4→5_ (black solid line), Go_4→6_ (gray line), and Go_5→7_ (black dotted line) for 480∼600th trials (i.e., from 20 trials before DA depletion to 100 trials after depletion) averaged across 20 simulations. The black and gray solid lines plot the same results that were shown in Fig. 6*Bf* with magnification. ***B***, Effective TD-RPE (i.e., post-DA-depletion TD-RPE was multiplied by 0.25, which was the assumed factor for size reduction of value increment due to DA depletion) after taking Go_4→6_ (left), Go_4→5_ (middle), and Go_5→7_ (right). The top panels show the results of individual simulation runs, where the white-red color indicates the magnitude of the effective TD-RPEs in reference to the rightmost color bar, and the black indicates the trials in which the corresponding action was not taken. The bottom panels show the trial-by-trial average of the effective TD-RPEs across simulations where the corresponding action was taken. The black thick line and the gray thin lines indicate the mean ± SEM of those simulations. ***C***, The ratio of choosing the HD arm (***a***), the latency (number of time steps) of reaching the T-junction (State 4; ***b***), and the mean effective TD-RPE per trial averaged over each successive 10 trials (***c***) until the 2000th trials in the cases where the obtained-reward-representing input to DA neurons gradually increases up to 2, 2.5, 3, 3.5, or 4 times of the original after DA depletion. The black thick line and the gray thin lines indicate the mean ± SEM of 20 simulations, and the vertical dotted line indicates the onset of DA depletion.

**Figure 8.**
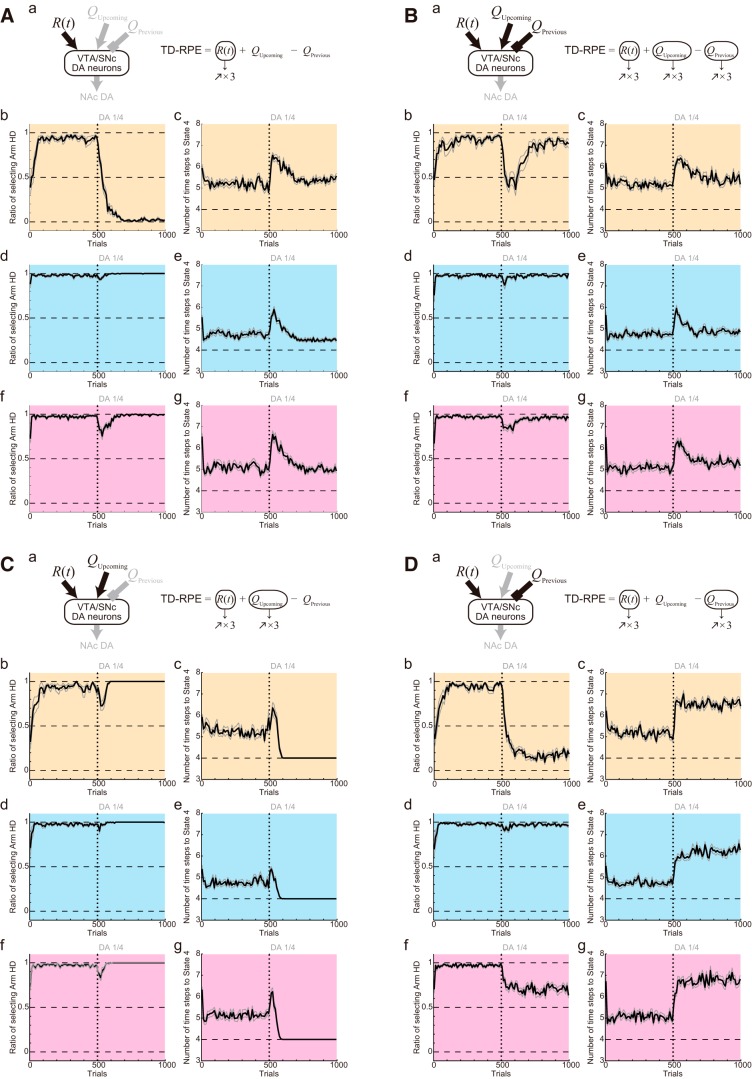
Results of the simulations with different assumptions. ***A***, Results with the assumption that the gain of the obtained-reward-representing input to DA neurons (black arrows in ***a***) gradually increases up to three times of the original after DA depletion. Panels ***b–g*** show the ratio of choosing the HD arm (***b***, ***d***, ***f***) and the latency (number of time steps) of reaching the T-junction (State 4; ***c***, ***e***, ***g***) in Conditions 1 (***b***, ***c***), 2 (***d***, ***e***), and 3 (***f***, ***g***). The black thick line and the gray thin lines indicate the mean ± SEM of 20 simulations. The same configurations are used in ***B–D***, except that in ***C***, 5 (***b***, ***c***), 2 (***d***, ***e***), and 2 (***f***, ***g***) simulation runs where action values became extremely large (action value larger than 100 times of the size of the large reward was detected) were omitted from the calculation of the mean and SEM. ***B***, Results with the assumption that the gain of all the three inputs that constitute the TD-RPE gradually increases up to three times of the original after DA depletion. ***C***, Results with the assumption that the gain of the obtained-reward-representing input and the upcoming-value-representing input gradually increases up to three times of the original after DA depletion. ***D***, Results with the assumption that the gain of the obtained-reward-representing input and the previous-value-representing input gradually increases up to three times of the original after DA depletion.

We explored how the discrepancy between the experimental and simulation results could be resolved by extending the model. It has been shown that DA depletion causes an increase in the neural activity in the pedunculopontine nucleus (PPN; Breit et al., 2001; Zhang et al., 2008; Geng et al., 2016), where separate populations of neurons were shown to represent obtained reward and expected reward values (Okada et al., 2009). Either or both populations have been proposed to contribute to the calculation of TD-RPE,

$$TD\text{-}RPE\left(t\right)=R\left(t\right)+Q_{\text{Upcoming}}-Q_{\text{Previous}},$$

in DA neurons by providing the information of the obtained reward [*R*(*t*); Kawato and Samejima, 2007; Okada et al., 2009; Morita et al., 2012], the value of previous action/state (–*Q_Previous_*; Cohen et al., 2012), and/or the value of upcoming action/state (*Q_Upcoming_*; Kawato and Samejima, 2007; Okada et al., 2009) via direct excitatory projections and/or indirect projections. In reference to these findings and suggestions, we considered extended models in which the gain of one or more terms of TD-RPE was assumed to gradually increase after DA depletion. DA depletion was assumed to cause a reduction of the size of TD-RPE–dependent value-update to a quarter of the original size (i) only when TD-RPE was nonnegative, and in separate sets of simulations, (ii) regardless of whether TD-RPE was nonnegative or negative. In the simulations shown in Figs. 3, 5, 6, 7, 8*A–C*, *Dd–g*, and S1 and the gray lines in Fig. 15, TD-RPE was always nonnegative and thus results for i and those for ii should be the same; practically, results for i were used to plot Fig. 3, 5, 6, 7, and 8*A–C*, *Dd–g* , whereas results for ii were used to plot Fig. S1 and the gray lines in Fig. 15. In the simulations shown in Figs. 8*Dbc* and 9, results for ii are shown (results for i can be obtained by using the codes uploaded in the ModelDB).

**Figure 9.**
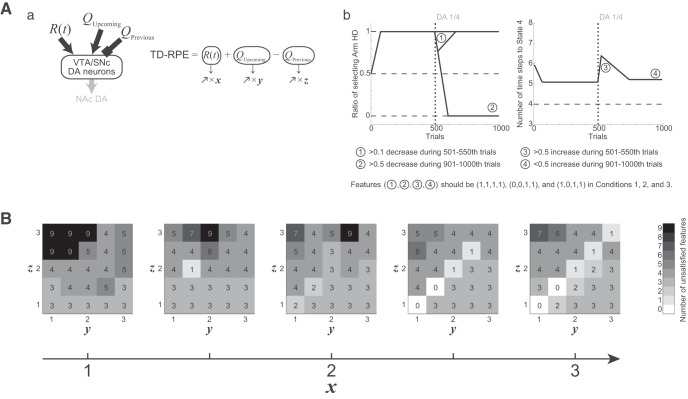
Systematic exploration of the possible secondary effects of DA depletion that best explain the experimental results. ***A***, ***a***, The gains of the inputs representing the obtained reward, upcoming value, and previous value were assumed to increase up to *x*, *y*, and *z* times, respectively, after DA depletion, and simulations of Conditions 1, 2, and 3 were conducted with *x*, *y*, and *z* systematically varied. ***b***, Criteria set to evaluate the simulation results by considering four features: for the ratio of choosing the HD arm (left panel), (1) >0.1 decrease during 501–550th trials and (2) >0.5 decrease during 901–1000th trials, and for the latency (number of time steps) of reaching the T-junction (State 4; right), (3) >0.5 increase during 501–550th trials and (4) <0.5 increase during 901–1000th trials (on average across trials and completed simulation runs). Simulation results were regarded as similar to the experimental results if the abovementioned features—1, 2, 3, and 4 were (1,1,1,1), (0,0,1,1), and (1,0,1,1), where 1 and 0 represent satisfied and unsatisfied—for Conditions 1, 2, and 3, respectively. ***B***, Number of features (1–4 above across the three conditions, in total 12) that were not satisfied in each set of simulation results with particular *x* (five panels), *y* (horizontal axes), and *z* (vertical axes).

### Simulation assuming the post-depletion increase of PPN input representing the obtained reward

We first considered a model in which the gain of the obtained-reward term [*R*(*t*)] selectively increases after DA depletion (Fig. 4), in accordance with some proposals (Morita et al., 2012). Specifically, we assumed that the gain gradually increases for 200 trials after DA depletion up to twice of the original, and then reaches a plateau (Fig. 4*C*). This time course and the plateau level were determined in reference to experimental literature (see Materials and Methods for details). Fig. 5 shows the results of simulations of the T-maze experiments (Salamone et al., 1994; Cousins et al., 1996) by using this model. As shown in the figure, the experimental results that DA depletion drastically changed the preference in Condition 1 but not in Condition 2 were reproduced by this model, as well (Fig. 5*Bb*,*Cb*). The extended model also reproduced the weak, transient reduction in the preference for the HD arm in Condition 3 (Fig. 5*Db*), as well as the transient increase and the subsequent decrease in the latency (Fig. 5*B*–*D*, panels *c*) although the decrease was less prominent compared with the experimental results.

To understand the mechanisms of how the extended model could reproduce the experimental results, we looked at the evolutions of the action values (Fig. 6). In Condition 1, the Go_4→5_ value was higher than the Go_4→6_ value before DA depletion (Fig. 6*Bb*). However, shortly after DA depletion (Fig. 6*Bc*), the Go_4→5_ value severely degraded and became smaller (although slightly) than the Go_4→6_ value (see also Fig. 6*Bf*), explaining the drastic change in the choice preference (Fig. 5*Bb*). This occurred because Go_4→5_ was separated from the rewarded state (State 7) and thus its value suffered the effect of DA depletion doubly. Specifically, the Go_4→5_ value was updated according to TD-RPE that contained the Go_5→7_ value, which was also updated according to TD-RPE, and both of these TD-RPE–dependent updates were affected by DA depletion. Indeed, although TD-RPE after taking Go_5→7_ should benefit from the increase in the gain of the obtained-reward term, such a benefit could not immediately, nor fully, compensate for the depletion effect, and thus the Go_5→7_ value decreased after depletion (Fig. 7*A*, right, black dotted line). Therefore, update of the Go_4→5_ value suffered from this decrease of the Go_5→7_ value, as well as the direct effect of depletion on TD-RPE after taking Go_4→5_, resulting in the even more severe decrease than the Go_5→7_ value (Fig. 7*A*, right, black solid line). In contrast, Go_4→6_ was next to the rewarded state (State 6) and so its value suffered the effect of DA depletion only singly. In fact, the Go_4→6_ value increased sometime after DA depletion (Fig. 7*A* right, gray line). This is because the severe decrease of the Go_4→5_ value caused less frequent choices of Go_4→5_ (i.e., the HD arm) and in turn more frequent choices of Go_4→6_ (the LD arm), and thereby the Go_4→6_ value became more frequently updated according to TD-RPE so that the balance between value-update and value-decay was shifted.

Fig. 7*B* shows effective TD-RPE, i.e., TD-RPE whose post-DA-depletion part was multiplied by one-quarter (because DA depletion was assumed to reduce the size of TD-RPE–dependent value update to one-quarter, as mentioned above) after taking Go_4→6_, Go_4→5_, or Go_5→7_. As shown in the right panel, the effective TD-RPE after taking Go_5→7_ once decreased after DA depletion, but subsequently increased again. This subsequent increase occurs because of a combination of the increase in the gain of the obtained-reward term and the decrease of the Go_5→7_ value, i.e., increase in the gap between the Go_5→7_ value and the obtained-reward term. Notably, although the effective TD-RPE after taking Go_5→7_ turned to increase, the Go_5→7_ value continued to decrease as seen above (Fig. 7*A*), because the frequency that Go_5→7_ was taken (i.e., the frequency of HD choice) went down (Fig. 5*Bb*; also in Fig. 7*B*, top panels) and the balance between value-update and value-decay shifted. In this way, the value-decay critically underlies the preference reversal: indeed, preference reversal did not occur when the decay rate was set to 0.001 instead of 0.01 [data not shown, but can be seen in the ModelDB (Fig. S1)].

In Condition 2 (Fig. 6*C*), the decrease of the Go_4→5_ value after DA depletion was much less prominent because Go_4→5_ was next to the rewarded state, explaining that the preference did not largely change (Fig. 5*Cb*). In Condition 3 (Fig. 6*D*), Go_4→5_ was again separated from the rewarded state, and so the Go_4→5_ value severely degraded after DA depletion. However, this time the Go_4→5_ value remained larger than the Go_4→6_ value, which was 0 because Go_4→6_ led to no reward (Fig. 6*Df*), explaining that the preference for the HD arm was weakened but not reversed to the LD arm (Fig. 5*Db*). Then, as the gain of the obtained-reward-representing input to DA neurons gradually increased as assumed, the values of Go_5→7_ and Go_4→5_ also gradually increased (Fig. 6*Dc–e*). The difference between the values of Go_4→5_ and Go_4→6_ thereby increased again (Fig. 6*Df*), explaining that the preference for the HD arm eventually recovered (Fig. 5*Db*).

Next, to consider the latency to reach the T-junction (State 4), we looked at the values of actions from the start to State 4. Before DA depletion, there were large value-contrasts between Go (arrows in Fig. 6*Bb*,*Cb*,*Db*) and Stay (circles) in all the three conditions (see, e.g., Go_3→4_ and Stay_3→3_ in Fig. 6*Bb*,*Cb*,*Db*; see also the pink and purple lines in Fig. 6*Bf*,*Cf*,*Df*). However, these value-contrasts degraded shortly after DA depletion, as shown in the figures. This degradation should cause an increase of the probability to choose Stay, and thereby explains the increase of the latency (Fig. 5*Bc*,*Cc*,*Dc*). Subsequently, as the gain of the obtained-reward term increased, value-contrasts between Go and Stay became reshaped, explaining the subsequent decrease of the latency. This mechanism suggested that the insufficient prominence in the subsequent latency decrease in the simulation results compared with the experimental results could be resolved if the gain of the obtained-reward term was further increased.

This was indeed confirmed, as shown in Fig. 7*Cb*. Specifically, when the gain increased up to 3 or 3.5 times of the original, the average latency of reaching the T-junction for 991∼1000th trials, as well as the average for 1991∼2000th trials, did not significantly differ from the average for 491∼500th trials (paired *t* test; *p* > 0.1). On the other hand, reversal of the preference in the arm choice could still occur in these cases (Fig. 7*Ca*), although it did not occur in some simulation runs, resulting in the relatively large standard errors. Fig. 7*Cc* shows the mean effective TD-RPE per trial averaged over each successive 10 trials (see Discussion).

### Simulation with different assumptions on the secondary effects of DA depletion

Next, we considered models assuming post-depletion increase of the gain of both upcoming-value and previous-value terms (*Q_Upcoming_* – *Q_Previous_*), or either the upcoming-value term (*Q_Upcoming_*) or the previous-value term (–*Q_Previous_*), in addition to the obtained-reward term. As a reference, Fig. 8*A* shows the case in which the gain of only the obtained-reward term increased up to three times of the original (different simulation runs with the same assumptions as those shown in Fig. 7*C*). Fig. 8*B* shows the results of simulations assuming the gain increase of all the three terms of TD-RPE. As shown in Fig. 8*Bb*, in Condition 1, DA depletion once drastically decreased the preference for the HD arm, but subsequently the preference increased again. This is inconsistent with the experimental results (Fig. 1*B*,*D*).

Fig. 8*C* shows the results of simulations assuming the gain increase of the obtained-reward and upcoming-value terms. With this assumption, in some of the simulation runs, action values became extremely large (action value larger than 100 times of the size of the large reward was detected) and simulation was quitted (5, 2, and 2 runs of 20 runs in Conditions 1, 2, and 3, respectively). Even in the other simulation runs, action values became quite large. Occurrence of such an inflation of action values was actually expected because, with this assumption, the upcoming-value term, whose gain increased after DA depletion, could not be well canceled out by the previous-value term, whose gain remained unchanged. The choice and latency patterns in the simulations (Fig. 8*Cb–g*) significantly deviated from the experimental results: the extremely short latency after DA depletion is considered to reflect the inflation of action (Go) values.

Fig. 8*D* shows the results of simulations assuming the gain increase of the obtained-reward and previous-value terms. The choice and latency patterns (Fig. 8*Db–g*) look somewhat similar to those observed in the simulations without assuming secondary effects (Fig. 3). In particular, the DA depletion-induced increase in the latency (Fig. 8*Dc*,*e*,*g*), as well as the decrease in the preference for the HD arm in Condition 3 (Fig. 8*Df*), were persistent rather than transient as observed in the experiments (Fig. 1*C*–*E*).

As so far shown, the experimentally observed behavioral results of DA depletion in the T-maze experiments could be reproduced when the gain of only the obtained-reward term increased after DA depletion but not when the other assumptions were made. To more systematically explore the possible secondary effects of DA depletion that best explain the experimental results, next we assumed that the gains of the inputs representing the obtained reward, upcoming value, and previous value increased up to *x*, *y*, and *z* times, respectively, after DA depletion, and simulations of Conditions 1, 2, and 3 were conducted with the parameters *x*, *y*, and *z* systematically varied [simulation was quitted when action value larger than 100 times of the size of the large reward (i.e., extremely large) was detected]. We then set criteria to evaluate the simulation results by considering the following four features: for the ratio of choosing the HD arm (average across trials and completed simulation runs), (1) >0.1 decrease during 501–550th trials (i.e., soon after depletion) and (2) >0.5 decrease during 901–1000th trials, and for the latency (number of time steps) of reaching the T-junction (State 4; average across trials and completed simulation runs), (3) >0.5 increase during 501–550th trials and (4) <0.5 increase during 901–1000th trials. Simulation results were regarded as similar to the experimental results if the abovementioned features 1,2,3,4 were (1,1,1,1), (0,0,1,1), and (1,0,1,1), where 1 and 0 represent satisfied and unsatisfied for Conditions 1, 2, and 3, respectively, and the number of unsatisfied features, of 4 features/condition × 3 conditions = 12 features in total, were counted for each set of simulation results with particular *x*, *y*, and *z*; when all the simulation runs for a given condition were quitted due to extremely large action value, all the features were regarded to be unsatisfied. As a result of this systematic exploration (Fig. 9*B*), parameter sets (among tested ones) with which all the features were satisfied turned out to be *x* = 2.5 or 3 and *y* = *z* = 1 or 1.5. This result supports the possibility that the gain of the obtained-reward-representing input prominently increased after DA depletion, whereas the previous and upcoming values-representing inputs entailed no or mild gain increase.

### Simulation of the D2 receptor antagonism experiments

**Figure 10.**
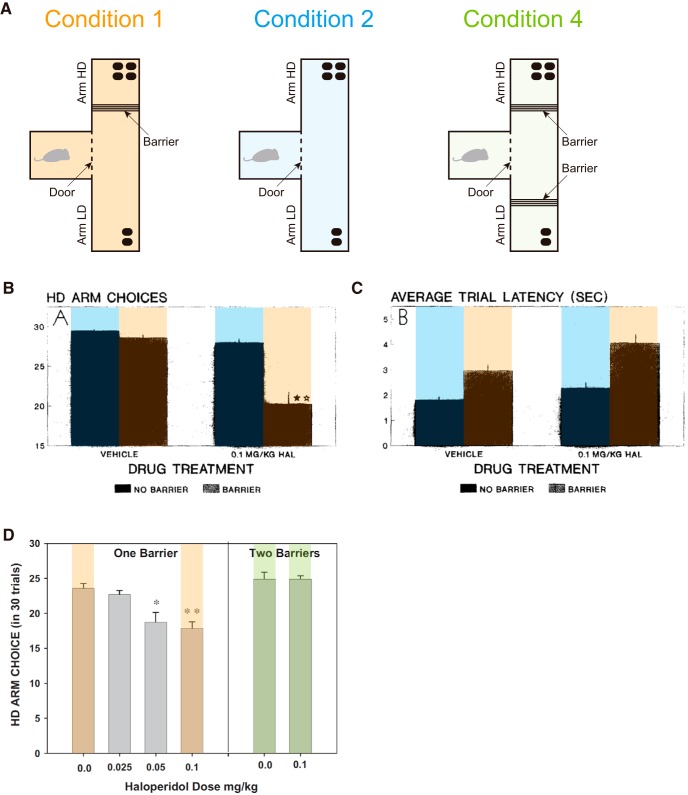
Experimental results on the effects of D2 receptor (D2R) antagonist in the effort-related T-maze choice task. ***B***, ***C***, and ***D*** were reprinted from Salamone et al. (1994), Copyright 1994, and Pardo et al. (2012), Copyright 2012, respectively, with permission from Elsevier; colors were added on the bars, also with permission. ***A***, Task conditions tested with D2R antagonist. In addition to Conditions 1 and 2, Condition 4, in which a physical barrier was placed in both the HD and LD arms, was tested. ***B***, ***C***, The ratio of selecting the HD arm (***B***) and the latency of start-door opening (***C***) in Condition 1 (orange-marked bars) and Condition 2 (blue-marked bars) in Salamone et al. (1994). The left two bars and the right two bars in each panel indicate the data for the rats that were injected with vehicle or D2R antagonist haloperidol, respectively. ***D***, The ratio of selecting the HD arm in Condition 1 (orange-marked bars) and Condition 4 (green-marked bars) in Pardo et al. (2012). The horizontal axis indicates the dose of D2R antagonist haloperidol injected into the mice.

In addition to the effects of DA depletion, effects of D2R antagonism have been examined in the T-maze experiments (Salamone et al., 1994). It was shown that injection of D2R antagonist haloperidol decreased HD choices in Condition 1, but not in Condition 2 (Fig. 10*B*), and also increased the latency in both Conditions 1 and 2 (Fig. 10*C*). Recent work (Pardo et al., 2012) examined the effects of haloperidol in mice in Condition 1 and a new condition, Condition 4, in which a barrier was placed in both arms (Fig. 10*A*), and showed that haloperidol decreased HD choices in Condition 1 but not in Condition 4 (Fig. 10*D*). We explored whether these results could also be explained by our model if possible effects of D2R antagonist on the circuit operation were incorporated.

**Figure 11.**
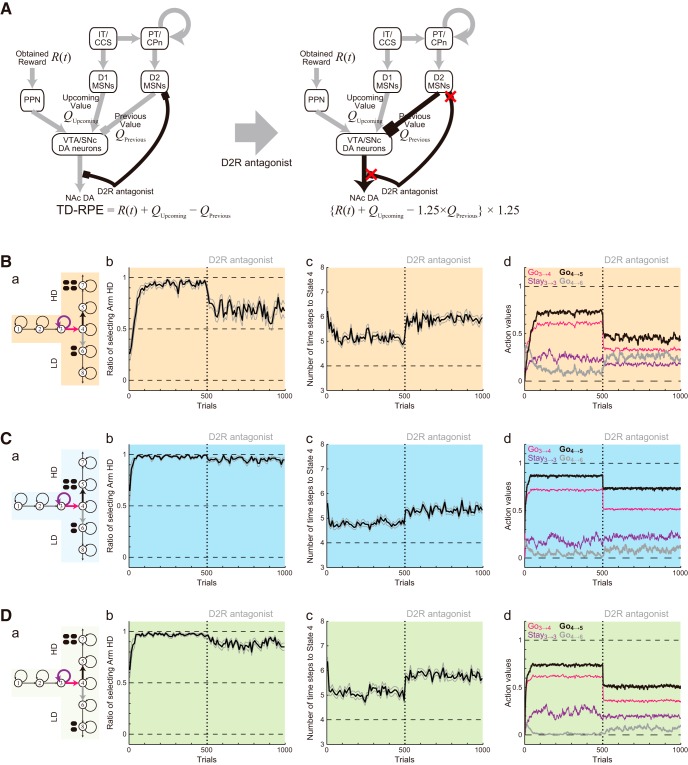
Results of the simulations of the effects of D2R antagonist with the assumption that D2R antagonist enhances DA/TD-RPE signaling and also amplifies the output of D2R-expressing striatal medium spiny neurons (D2 MSNs) that presumably encode the value of previous action. ***A***, Assumed effects of D2R antagonist. The antagonist was assumed to enhance DA/TD-RPE signaling, and also relieve D2R-mediated inhibition of D2 MSNs, which presumably encode the value of previous action (as illustrated) according to one hypothesis on the mechanism of TD-RPE calculation, named the CS-TD hypothesis (Morita et al., 2012, 2013; Morita, 2014; Morita and Kawaguchi, 2015). These presumed effects were incorporated into the model as an amplification (1.25 times) of TD-RPE–dependent value update and also an amplification (1.25 times) of the previous-value term in TD-RPE. ***B***, ***C***, ***D***, Simulation results for Conditions 1, 2, and 4. The configurations are the same as those in Fig. 5*B*,*C* and Fig. 6*Bf*,*Cf*. ***a***, Schematics of the simulated task conditions. ***b***, The ratio of choosing the HD arm. ***c***, The latency (number of time steps) of reaching the T-junction (State 4). ***d***, Trial-by-trial evolutions of the learned values of Go_3→4_, Stay_3→3_, Go_4→5_, and Go_4→6_.

Activation of D2Rs on the DA axons inhibits DA release, causing a negative feedback, and D2R antagonist relieves such an inhibition (Gonon and Buda, 1985) and also inhibits DA uptake (Benoit-Marand et al., 2011), causing an enhancement of DA signaling. We incorporated this into the model as an amplification of TD-RPE–dependent value update. D2Rs are also expressed in about half of the striatal medium spiny neurons (MSNs), while the other half of MSNs express D1Rs (Gerfen and Surmeier, 2011). Activation of D2Rs causes a reduction of the responsiveness of D2-MSNs (Gerfen and Surmeier, 2011), and D2R antagonist is considered to block such a reduction and thereby amplify the output of D2-MSNs. The roles of D2Rs or D2-MSNs in reward learning have been examined by using pharmacological (Pessiglione et al., 2006; Lee et al., 2015) and optogenetic (Kravitz et al., 2012) manipulations. Their results have suggested that administration of L-DOPA or D2R antagonist (haloperidol) resulted in differential magnitude of RPE (larger in the former) in humans (Pessiglione et al., 2006), while injection of D2R antagonist (eticlopride) into the dorsal striatum of monkeys resulted in a decrease in the inverse temperature (Lee et al., 2015), and also that stimulation of D2-MSNs induced transient punishment in mice (Kravitz et al., 2012). One hypothesis, the CS-TD hypothesis (Morita et al., 2012, 2013; Morita, 2014; Morita and Kawaguchi, 2015), posits that D2-MSNs represent the value of previous action/state and negatively impact the DA neurons via the indirect pathway of the basal ganglia (Fig. 11*A*, left). This hypothesis could potentially explain [as shown in Morita et al. (2013)] the abovementioned optogenetic results (Kravitz et al., 2012) although in a different way from the authors’ explanations. If this hypothesis holds, the presumable amplification of the D2-MSNs output by D2R antagonist should cause an amplification of the previous-value term (–*Q_Previous_*) of TD-RPE, which we incorporated into the model (Fig. 11*A*, right). In the results (Fig. 11*B*–*D*), D2R antagonist reduced the preference for the HD arm prominently in Condition 1 but much less prominently in Conditions 2 and 4, while increasing the latency in all the conditions. These results are at least partially in line with the experimental results (Fig. 10), although the absence of the effect in the choices in Conditions 2 and 4 was not reproduced.

To understand the underlying mechanisms, we looked at the action values. In Condition 1 (Fig. 11*Bd*), the Go_4→5_ value, referred to as *Q*(Go_4→5_), markedly decreased after the administration of D2R antagonist. This should be because TD-RPE generated after taking Go_4→5_ negatively shifted due to the presumed antagonist-induced amplification of the previous-value term [i.e., −*Q*(Go_4→5_)] so that *Q*(Go_4→5_) was negatively updated according to the TD-RPE. In contrast, as for TD-RPE generated after taking Go_4→6_, amplification of the previous-value term [i.e., − *Q*(Go_4→6_)] could cause only a weaker effect because *Q*(Go_4→6_) was smaller than *Q*(Go_4→5_), and thus marked decrease of *Q*(Go_4→6_) did not occur. The marked decrease of *Q*(Go_4→5_) but not of *Q*(Go_4→6_) led to the prominent decrease of the ratio of choosing Go_4→5_ (HD arm), which eventually resulted in a drastic shift in the balance between value-update and value-decay, causing an increase of *Q*(Go_4→6_). Also, D2R antagonist similarly caused a prominent decrease of *Q*(Go_3→4_), but not of *Q*(Stay_3→3_), explaining the increase in the latency.

**Figure 12.**
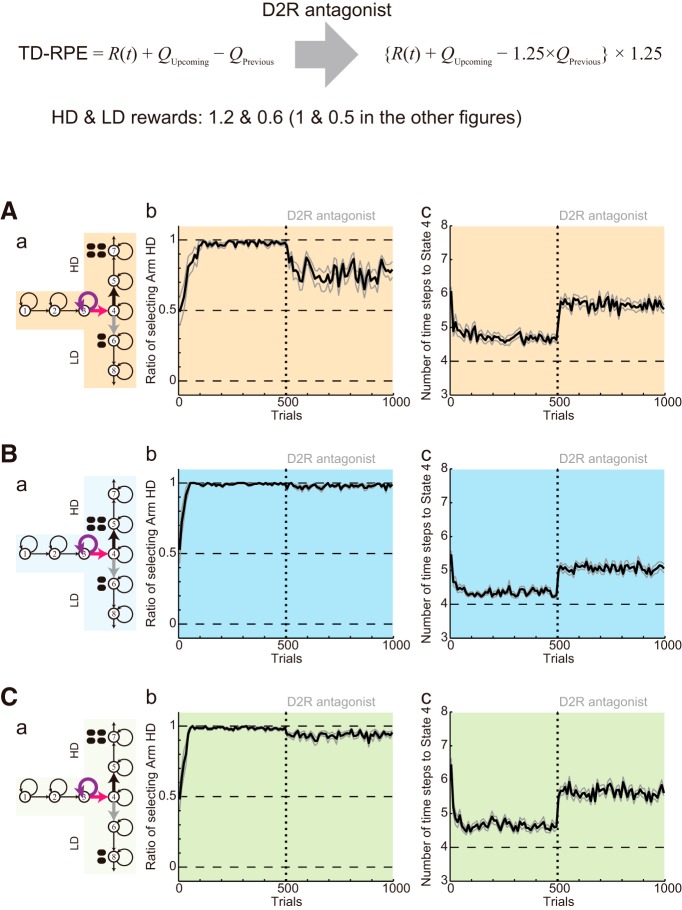
Results of the simulations of the effects of D2R antagonist, assuming the enhancement of DA/TD-RPE signaling and the amplification of the output of D2 MSNs (in the same manner as in Fig. 11), with the sizes of the rewards increased from the original ones (large = 1, small = 0.5) to large = 1.2, small = 0.6. ***A–C***, Simulation results for Conditions 1, 2, and 4. The configurations are the same as those in Fig. 11*B*,*C*,*Da–c*. ***a***, Schematics of the simulated task conditions. ***b***, The ratio of choosing the HD arm. ***c***, The latency (number of time steps) of reaching the T-junction (State 4).

In Conditions 2 and 4 (Fig. 11*Cd*,*Dd*), D2R antagonist caused a decrease of *Q*(Go_4→5_) similarly to Condition 1. However, because the difference between *Q*(Go_4→5_) and *Q*(Go_4→6_) before antagonist administration was larger than the case of Condition 1, a shift in the balance between value-update and value-decay was caused less prominently and therefore the change in the ratios of HD and LD choices was less prominent. This mechanism suggested that moderately increasing the magnitudes of HD and LD rewards in simulations might increase the initial difference between *Q*(Go_4→5_) and *Q*(Go_4→6_) in Conditions 2 and 4 and reduce the changes in the choice ratios, making the results closer to the experimental results. Indeed, this expectation was successfully confirmed (Fig. 12).

### Simulation of the D1 receptor antagonism experiments

**Figure 13.**
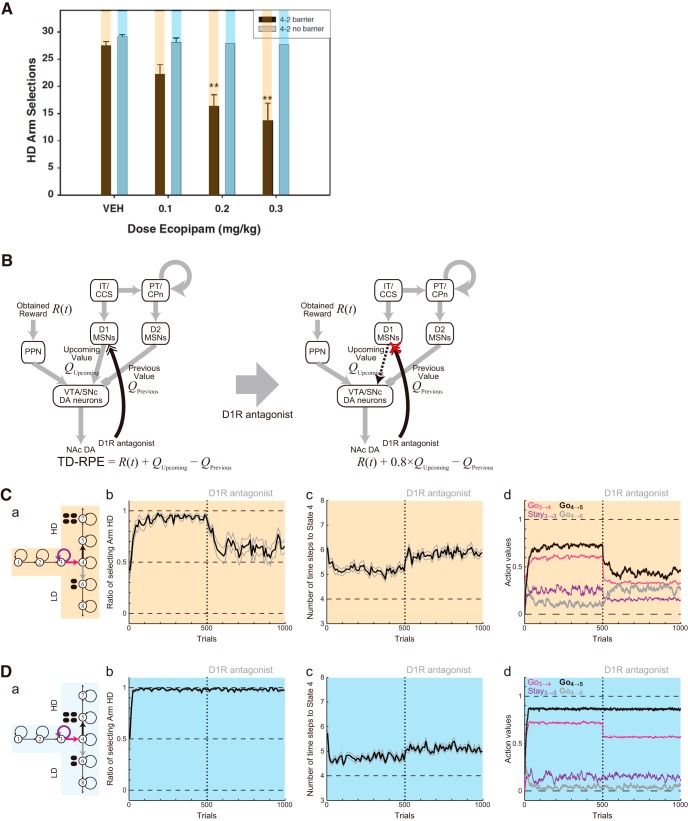
Experimental results on the effects of D1R antagonist, and simulation results with the assumption that D1R antagonist reduces the output of D1 MSNs that presumably encode the value of upcoming action. ***A*** was reprinted from Yohn et al. (2015), Copyright 2015, with permission from Elsevier; colors were added on the bars, also with permission. ***A***, The number of selecting the HD arm, out of a total of 30 trials, in Condition 1 (orange-marked bars) and Condition 2 (blue-marked bars) in Yohn et al. (2015). The horizontal axis indicates injection of vehicle (VEH) or the dose of D1R antagonist ecopipam. ***B***, Assumed effect of D1R antagonist. The antagonist blocks D1R-mediated upregulation of D1 MSNs, which presumably encode the value of upcoming action (as illustrated) according to the CS-TD hypothesis. This effect was incorporated into the model as a reduction (to 0.8 times of the original) of the upcoming-value term of TD-RPE. ***C***, ***D***, Simulation results for Conditions 1 and 2. The configurations are the same as those in Fig. 11*B*,*C*. ***a***, Schematics of the simulated task conditions. ***b***, The ratio of choosing the HD arm. ***c***, The latency (number of time steps) of reaching the T-junction (State 4). ***d***, Trial-by-trial evolutions of the learned values of Go_3→4_, Stay_3→3_, Go_4→5_, and Go_4→6_.

It has been shown that D1R antagonism also specifically impaired the choice of high-cost, large-reward option (Fig. 13*A*) and increased the latency of start-door opening (Yohn et al., 2015). D1Rs are expressed in the other half of MSNs, and activation of D1Rs causes an enhancement of the responsiveness of D1-MSNs (Gerfen and Surmeier, 2011). D1R antagonist is considered to block such an enhancement and thereby reduce the output of D1-MSNs. The CS-TD hypothesis, introduced above, posits that D1-MSNs represent the value of upcoming action/state and positively impact the DA neurons via the direct pathway of the basal ganglia (Fig. 13*B*, left). If this is the case, the presumable reduction of the D1-MSNs output by D1R antagonist should cause a reduction of the upcoming-value term (+*Q_Upcoming_*) of TD-RPE. We incorporated such a reduction into the model (Fig. 13*B*, right; see Materials and Methods for details) and conducted simulations. In the results (Fig. 13*C*,*D*), D1R antagonist reduced the preference for the HD arm prominently in Condition 1 (Fig. 13*Cb*) but almost not in Condition 2 (Fig. 13*D*b), while increasing the latency in both conditions (Fig. 13*Cc*,*Dc*). These results are in line with the experimental results. Looking at the action values in Condition 1, *Q*(Go_4→5_) and *Q*(Go_3→4_) prominently decreased after antagonist administration (Fig. 13*Cd*,*Dd*). These decreases, which explain the impairment in HD choices and the increase in the latency, are considered to occur because the assumed reduction of the upcoming-value term (*Q_Upcoming_*) of TD-RPE negatively shifted TD-RPE.

### Simulation of the DA depletion experiments, with alternative assumptions for depletion effects

**Figure 14.**
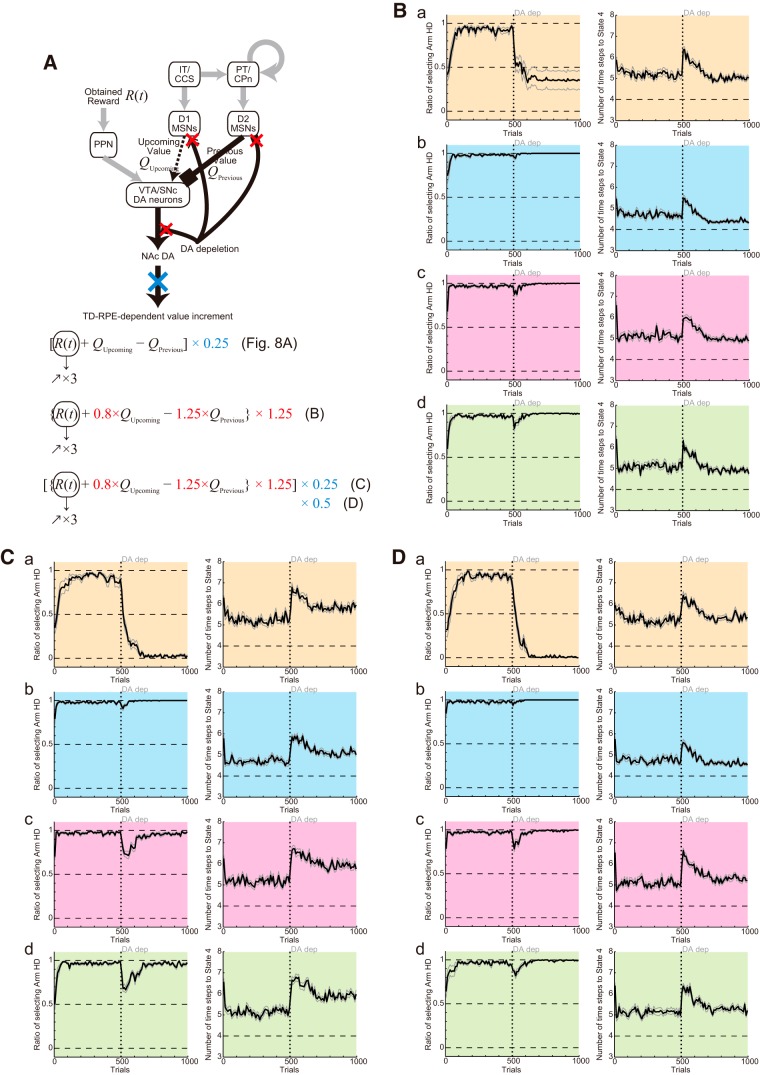
Simulation of the DA depletion experiments, with alternative assumptions for depletion effects. ***A***, DA depletion was assumed to cause a reduction of the size of TD-RPE–dependent value update (indicated by the blue cross in the schematic and blue terms in the equations) in the simulations shown in Fig. 8*A*, but DA depletion can instead or in addition cause modulations of the responsiveness of MSNs and DA axons expressing D1Rs/D2Rs, i.e., effects similar to those assumed to be caused by D1R/D2R antagonisms (red crosses in the schematic and red terms in the equations). ***B***, Results of simulations for Conditions 1–4 (a–d), assuming that DA depletion causes modulations of the responsiveness of MSNs and DA axons expressing D1Rs/D2Rs, as well as a gradual increase of the gain of the obtained-reward input, but not a reduction of the size of TD-RPE–dependent value update. ***C***, Results of simulations for Conditions 1–4 (a–d), assuming that DA depletion causes both modulations of the responsiveness of MSNs and DA axons expressing D1Rs/D2Rs and a reduction of the size of TD-RPE–dependent value update, as well as a gain increase of the obtained-reward input. ***D***, Same as ***C*** except for assuming that the DA-depletion-induced reduction of the size of TD-RPE–dependent value update was milder: 50%, rather than 25%, of the original. The configurations in ***B–D*** are the same as those in the previous figures.

In the above, we simulated DA depletion experiments with the assumption that DA depletion causes quarterization of TD-RPE–dependent value increment or value update (Fig. 14*A*, the first equation). However, DA depletion could instead, or in addition, causes modulations of the responsiveness of MSNs and DA axons expressing D1Rs/D2Rs, similarly to D1R/D2R antagonisms. We tested this possibility by performing separate sets of simulations assuming the same effects as assumed in the simulations of D1R/D2R antagonisms in the above (Fig. 14*A*, second equation), or those effects in addition to quarterization or halving of TD-RPE–dependent value update (regardless of whether TD-RPE was nonnegative or negative; Fig. 14*A*, third equation); the gain increase of the obtained-reward term of TD-RPE was also assumed in all the cases. In the results (Fig. 14*B*–*D*), the choice and latency patterns were largely in line with the experimental results (Fig. 1), except that eventual decrease in the latency was less prominent in the case assuming both modulations of MSNs/DA axons and quarterization of value update (Fig. 14*C*). These results indicate that both of the assumed effects of DA depletion, i.e., modulations of MSNs/DA axons and (mild) size reduction of value update, might contribute to the experimentally observed behavioral effects.

### Predictions of the model

**Figure 15.**
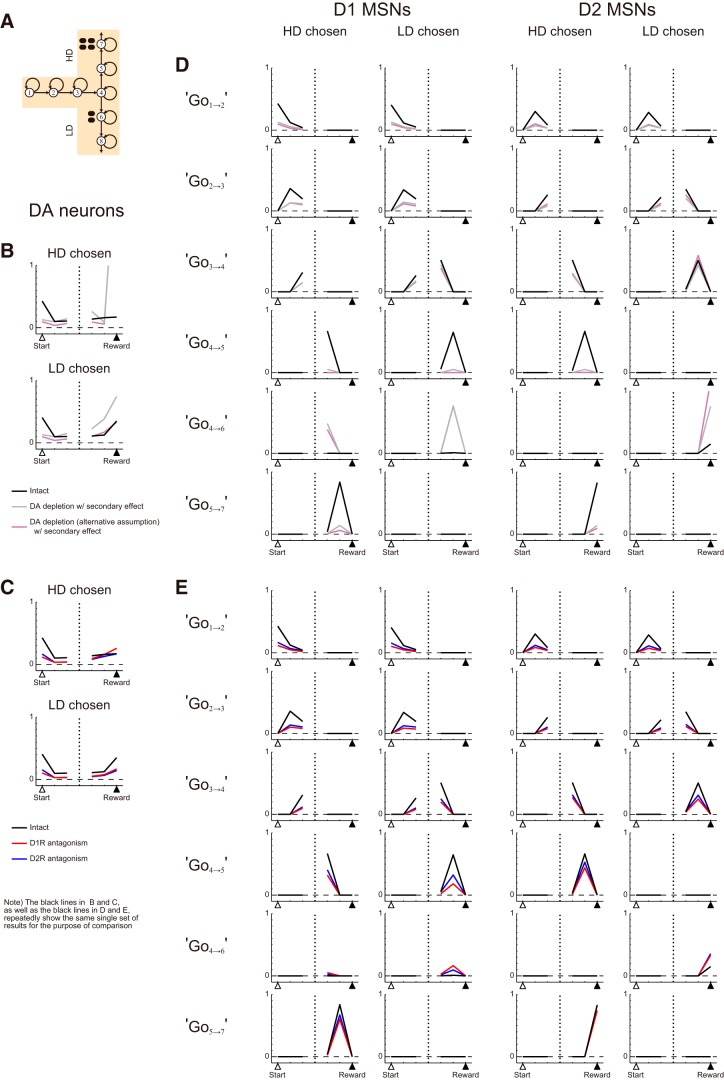
Model’s predictions about activity patterns of DA neurons, D1 MSNs, and D2 MSNs in the cases without or with DA manipulations. ***A***, Schematic illustration of Condition 1 of the T-maze task. ***B***, Predicted activity pattern of DA neurons without or with DA depletion. The black lines indicate the cases without DA depletion. The gray lines indicate the cases with DA depletion modeled as a reduction of the size of TD-RPE–dependent value update to 25% of the original, and also with the secondary effect (increase in the gain of the obtained-reward-representing input to DA neurons up to three times). The purple-gray lines indicate the cases with DA depletion modeled in an alternative way, i.e., as a reduction of the size of TD-RPE–dependent value update to 50% of the original and modulations of the responsiveness of MSNs and DA axons expressing D1Rs/D2Rs, and also with the secondary effect (the same assumption as made in Fig. 14*D*). The lines indicate the mean activity in the trials in which the HD arm was chosen (panels indicated as HD chosen) or the LD arm was chosen (LD chosen) during 251–500 trials (in the cases without DA manipulations) or 751–1000 trials (i.e., 251–500 trials from the onset of DA manipulations, in the cases with DA manipulations) in Condition 1 at the time steps aligned at the start (open triangle) or the reward (filled triangle), averaged across 20 simulations. ***C***, Predicted activity pattern of DA neurons without or with DA antagonism. The black lines indicate the cases without DA antagonism and are identical to the black lines in ***B***, i.e., repeatedly show the same single set of results for the purpose of comparison. The red and blue lines indicate the cases with D1R antagonism or D2R antagonism, respectively. ***D***, Predicted activity patterns of MSNs corresponding to Go actions without or with DA depletion. The black lines indicate the cases without DA depletion, and the gray lines and purple-gray lines indicate the cases with DA depletion modeled in the two ways as in ***B***. ***E***, Predicted activity patterns of MSNs corresponding to Go actions without or with DA antagonism. The black lines indicate the cases without DA antagonism and are identical to the black lines in ***D***, i.e., repeatedly show the same single set of results for the purpose of comparison. The red lines and blue lines indicate the cases with D1R antagonism or D2R antagonism, respectively.

Because our model describes the temporal change in the activity of DA neurons and striatal MSNs, our model provides predictions about the pattern of neural activity and how it is affected by DA manipulations. The black lines in Fig. 15*B* show the predicted activity pattern of DA neurons, at the time steps aligned at the times of start and reward, after learning has settled down (averaged over 251∼500 trials) in Condition 1 (Fig. 15*A*) without DA manipulations: the two panels separately show the cases where the HD or LD arm was chosen. Although learning has settled down, DA neurons are predicted to show activity not only at the time of start but also at the time of reward. Such sustained DA signals have been experimentally observed (Howe et al., 2013; Hamid et al., 2016), and our model successfully explains such signals by virtue of the value-decay, as we have previously explained (Morita and Kato, 2014; Kato and Morita, 2016). Our model further predicts that DA neuronal activity at the time of reward is higher when the LD arm is chosen than when the HD arm is chosen (*t* test; *p* < 10^−9^). This is because the LD arm is not frequently chosen, so the value-decay is relatively predominant compared to the value-update, resulting in the Go value preceding the reward remaining small and TD-RPE remaining large.

The gray lines and purple-gray lines in Fig. 15*B* indicate the predicted activity pattern of DA neurons in the case with DA depletion, averaged over 751–1000 trials, i.e., 251–500 trials from the onset of DA depletion, with DA depletion modeled either as quarterization of TD-RPE–dependent value update (gray lines; same as in Fig. 8*A*) or as halving of value update and modulations of the responsiveness of MSNs and DA axons (purple-gray lines; same as in Fig. 14*D*); the gain increase of the obtained-reward-representing input was assumed in both cases. As shown in the figures, the model predicts that DA depletion decreases the DA neuronal activity at the start of trial, regardless of the way DA depletion is modeled. Meanwhile, predicted DA neuronal activity is lower at most timings when depletion is assumed to also cause modulations of the responsiveness of MSNs and DA axons (purple-gray lines). This is reasonable because the modulations of MSNs’ responsiveness are assumed to cause a negative shift in the net input to DA neurons. The red and blue lines in Fig. 15*C* indicate the predicted DA neuronal activity in the cases with D1R or D2R antagonism, respectively. As shown in the figures, in both cases, the activity at the times of start and LD-reward is predicted to be smaller than the intact case.

The black lines in Fig. 15*D* indicate the predicted activity pattern of D1 MSNs (left panels) and D2 MSNs (right panels) representing the value of a single Go action in the cases without DA manipulations. As shown in the figure, there are predicted to be neurons with activity peaking at various time points, in both D1 MSNs and D2 MSNs populations. The gray and purple-gray lines in Fig. 15*D* indicate the cases with DA depletion, modeled in the two different ways, as well as the secondary effect, and the red and blue lines in Fig. 15*E* indicate the cases with D1R or D2R antagonism, respectively. As shown in these figures, DA depletion and antagonisms are predicted to down-regulate the activity of MSNs in most cases, although the activity of MSNs representing the value of Go_4→6_ is up-regulated by the manipulations, corresponding to the shift in the choice preference toward the LD arm.

### Potential explanation of apparently contradictory results

**Figure 16.**
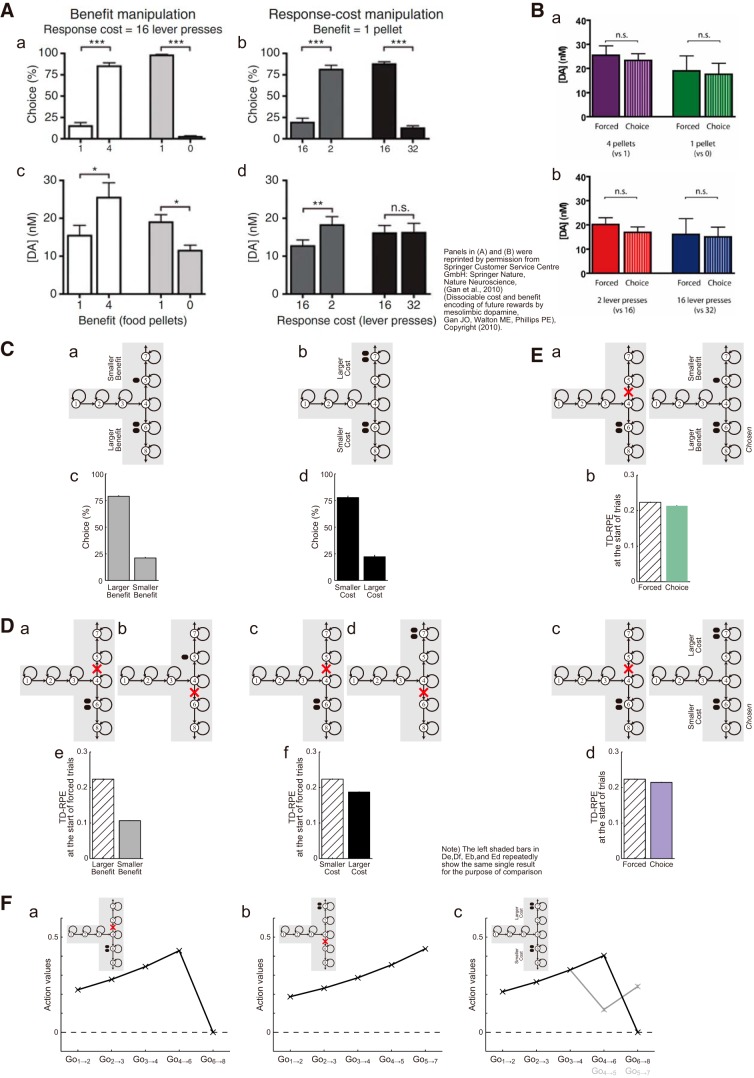
Potential explanation of the experimentally observed DA’s limited encoding of cost level in a different task paradigm by the model. Panels in ***A*** and ***B*** were reprinted by permission from Springer Customer Service Center GmbH: Springer Nature, Nature Neuroscience (Gan et al., 2010; Dissociable cost and benefit encoding of future rewards by mesolimbic dopamine, Gan JO, Walton ME, Phillips PE, *Nat Neurosci* 13:25–27), Copyright 2010. ***A***, ***a***, ***b***, The choice rate of options associated with different sizes of benefit (horizontal axis) with the cost equalized (***a***), or options associated with different amounts of cost (horizontal axis) with the benefit equalized (***b***). ***c***, ***d***, Cue-evoked nucleus accumbens DA in forced trials with different sizes of benefit (***c***) or different amounts of cost (***d***). ***B***, Comparison of cue-evoked DA between forced trials (left bars of ***a***, ***b***) and choice trials where high-utility option was chosen (right bars of ***a***, ***b***) with different amounts of benefit (***a***) or cost (***b***). ***C***, ***a***, Schematics of the simulated benefit-based choice trials, in which larger or smaller benefit (size 0.5 or 0.25) could be obtained by first reaching State 6 or 5, respectively. ***b***, Schematics of the simulated cost-based choice trials, in which equal benefit (size 0.5) could be obtained by first reaching State 6 or 7. ***c***, ***d***, The choice rate of arms with different sizes of benefit (***c***) or different amounts of cost (***d***) in the simulated task. The bar height indicates the mean choice rate for 251∼500 trials averaged across 20 simulations, and the error bar indicates the SEM of 20 simulations (these are applied also to panels ***D***, ***e***, and ***f***, and ***E***, ***b*** and ***d***). ***D***, ***a***, ***b***, Schematics of the simulated forced trials with larger benefit (***a***) or smaller (***b***) benefit, where the red crosses indicate that Go_4→5_ or Go_4→6_ was disabled, respectively (the same is applied also to ***c***, ***d***). ***c***, ***d***, Schematics of the simulated forced trials with smaller cost (***c***) or larger cost (***d***). ***e***, ***f***, TD-RPE generated at the start of a trial in the simulated forced trials with different sizes of benefit (***e***) or different amounts of cost (***f***). Notably, whereas choice trials and forced trials with one of the two options in the choice trials were intermingled, or more specifically, blocks of 4 forced trials and subsequent 4 choice trials were repeated in sessions where DA recording was made in the experiments (Gan et al., 2010), we simulated different types of forced trials separately from each other and also separately from choice trials. Also notably, in the model, the larger-benefit forced trials (***D***, ***a***) were identical to the smaller-cost forced trials (***D***, ***c***), and thus we conducted only a single set of simulations that corresponded to both types of forced trials and therefore the left shaded bars in ***e*** and ***f*** are identical, i.e., repeatedly show the same single simulation result for the purpose of comparison. ***E***, ***a***, ***c***, Schematics of the simulated forced trials and choice trials. ***b***, ***d***, The right bars indicate TD-RPE at the start of the simulated benefit-based (***b***) or cost-based (***d***) choice trials where high-utility option was chosen. The left shaded bars indicate TD-RPE at the start of the simulated larger-benefit/smaller-cost forced trials, and are both identical to the left bars in ***D***, ***e*** and ***f***, i.e., repeatedly show the same single simulation result for the purpose of comparison. ***F***, Learned values of Go actions in the simulated smaller-cost (***a***) or larger-cost (***b***) forced trials and cost-based choice trials (***c***). The lines indicate the mean for 251∼500 trials averaged across 20 simulations, and the error bars indicate the SEM of 20 simulations. In ***c***, the gray line indicates the values of Go_4→5_ and Go_5→7_.

Finally, we examined whether our model could also explain apparently contradictory results in the literature. Whereas there are a number of studies suggesting the involvements of DA in effort-related choices, DA measurement during a task with benefit-based or cost-based choices (Gan et al., 2010) revealed that DA evoked at the presentation of a cue predicting the level of benefit or cost encoded the benefit level well, but the cost level only in a limited manner. Specifically, rats were trained to make either a benefit (reward)-based choice, choosing a cue associated with large or small benefit with the cost (number of lever presses) equalized, or a cost (effort)-based choice, choosing a cue associated with small or large cost with the benefit equalized. Rats successfully learned to preferentially choose a large-benefit cue (Fig. 16*Aa*), as well as a small-cost cue (Fig. 16*Ab*). However, cue-evoked nucleus accumbens (NAc) DA in forced trials, where only a single cue was presented, well encoded the benefit level (Fig. 16*Ac*) but not the cost level (Fig. 16*Ad*): cost encoding was limited to the case with low cost and it diminished over training.

We qualitatively simulated this task by our model, having different sizes of benefits (size 0.5 and 0.25) on the states near the T-junction to simulate benefit-based choices (Fig. 16*Ca*) or the equal benefits (size 0.5) on the states near to and distant from the T-junction to simulate cost-based choices (Fig. 16*Cb*). With the same set of parameters (learning rate, inverse temperature, value-decay, and no temporal discounting) used so far, simulated subjects learned to preferentially choose the larger-benefit arm (Fig. 16*Cc*) and the smaller-cost arm (Fig. 16*Cd*) to a comparable level (*t* test, *p* = 0.487). Next, we simulated forced trials by disabling Go action from the T-junction to an arm corresponding to unavailable option (Fig. 16*Da–d*). Notably, whereas choice trials and forced trials were intermingled in the experiments, or more specifically, blocks of four forced trials and subsequent four choice trials were repeated in sessions where DA recording was made in the experiments (Gan et al., 2010), we simulated different types of forced trials separately from each other and also separately from choice trials. Also notably, in the model, the larger-benefit forced trials (Fig. 16*Da*) were identical to the smaller-cost forced trials (Fig. 16*Dc*), and thus we conducted only a single set of simulations that corresponded to both types of forced trials. We looked at TD-RPE generated at the start of a trial, after learning has settled down separately for each type of forced trials, as a counterpart of DA evoked at the presentation of a cue, and found that the TD-RPE differed prominently between cases with different sizes of benefit (Fig. 16*De*) but more mildly between cases with different amounts of cost (Fig. 16*Df*). This limited encoding of cost levels as compared to benefit-level encoding, despite the comparable behavioral preference, resembles the experimental results (Fig. 16*A*) to a certain extent, although the across-session diminishment of cost encoding is not explained in the model.

In our simulations, forced smaller-cost trials and forced larger-cost trials differed in the number of states from start to reward (Fig. 16*Dc*,*d*), and difference in the TD-RPE at the start between them corresponds to difference between the values of neighboring Go actions (Fig. 16*Fa*,*b*), which is shaped by the value-decay (Kato and Morita, 2016) and is relatively small. The reason that the difference in the cost level could nevertheless cause the prominent difference in the simulated choice trials is because the value-decay causes value-contrasts between well-chosen actions and less-chosen actions (Kato and Morita, 2016); i.e., in the simulated choice trials, the values of actions on a less frequently chosen arm are less frequently updated by TD-RPEs and thereby effectively decay more (Fig. 16*Fc*, gray line), amplifying the difference in the frequencies of arm choices. A key feature of our simulation of the task is that we simulated choice trials and each type of forced trials separately, as mentioned above. Notably, TD-RPE at the start of choice trials where high-utility option was chosen was close to TD-RPE in forced trials with the same option (Fig. 16*E*), appearing to resemble the experimental results (Fig. 16*B*), although in the simulations there were actually statistical differences because of small variances. Our results suggest that choice trials and forced trials might indeed be learned individually, at least to a certain extent, in the experiments, although they were intermingled, and the value of the action that imposes an extra cost significantly decayed only in the case where it needed not to be taken, i.e., in choice trials but not in forced trials, resulting in the observed apparently contradictory prominent cost avoidance in choice trials and DA’s limited cost encoding in forced trials.

## Discussion

**Figure 17.**
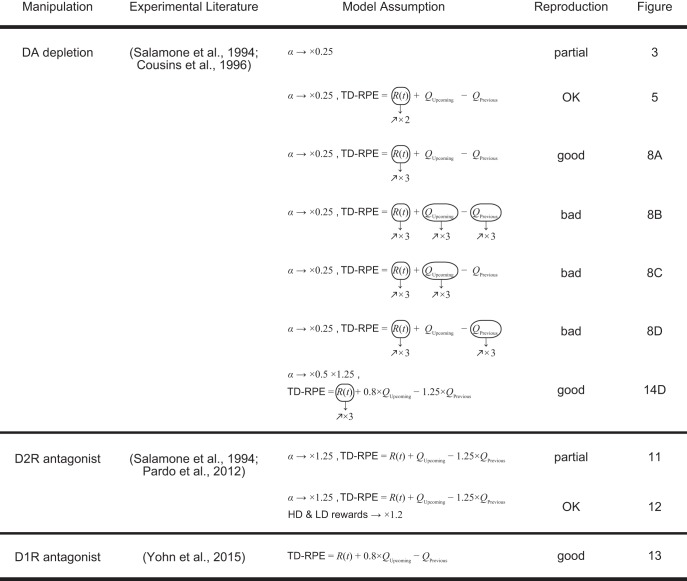
Summary on how the model could reproduce the effects of DA manipulations in the effort-related choice experiments.

We have shown that the effects of DA depletion in the effort-related choice experiments, namely, the impairment of reward-oriented effort exertion and the transient increase in the latency, could be explained by the model assuming DA’s role as TD-RPE and the decay of learned values, given that the gain of the obtained-reward-representing input to DA neurons increased after DA depletion. Such a gain increase is assumed to occur through a post-depletion increase of the activity of PPN neurons representing the obtained reward. We have also shown that the impairment of reward-oriented effort exertion by D1R or D2R antagonism could also be explained by the same model, given a proposed mechanism of TD-RPE calculation named the CS-TD hypothesis, in which the D1 and D2 pathways encode the values of actions with a temporal difference (see Fig. 17 for results summary and Table 1 for comparison with our previous studies). So far, while the results of DA depletion and antagonisms have been regarded as key evidence for DA’s involvements in effort-related choices, the underlying circuit mechanisms have remained unclear. Our results suggest that those pharmacological results might be at least partially explained from DA’s role as TD-RPE, for which the circuit mechanisms have now become clarified. Simultaneously, our results in turn suggest a way to take advantage of the rich pharmacological results to constrain hypotheses on the mechanisms of TD-RPE calculation.

**Table 1.** Evolution of models incorporating the decay/forgetting of learned values

Model	(Morita and Kato, 2014)	(Kato and Morita, 2016)	Present work
Mechanisms that have been incorporated			
Decay/forgetting of learned values	**✓**	**✓**	**✓**
Self-paced behavior as Go or Stay selection		**✓**	**✓**
Secondary effects of DA depletion			**✓**
Temporal difference in D1 and D2 pathways			**✓**
Phenomena that could be explained			
Sustained/ramping DA signals	**✓**	**✓**	**✓**
Slowdown by DA depletion		partial (not about temporariness)	**✓**
Effort impairment by DA depletion		partial (not about Condition 3)	**✓**
Effort impairment by D1 or D2 antagonism			**✓**

The mechanisms that have been incorporated, and the phenomena that could be explained, are shown for the present model and the previous models.

### NAc DA content and response vigor

In our simulations of DA depletion assuming quarterization of TD-RPE–dependent value update, the mean effective TD-RPE decreased after DA depletion, and then increased again, especially when the gain of the obtained-reward input to DA neurons was assumed to prominently increase, as shown in Fig. 7*Cc*. The time course of the subsequent increase of the mean effective TD-RPE looks similar to that of the subsequent decrease of latency (Fig. 7*Cb*). However, whereas the latency could return to the original level when the gain of the obtained-reward input increased up to 3 or 3.5 times, the mean effective TD-RPE remained much smaller than the original level. Given that the mean effective TD-RPE could correspond to neurochemically measured DA content, this simulation result could be in line with the experimental result reported in (Salamone et al., 1994) that the DA content in NAc in the DA-depleted rats was 20.3%∼23.7% of the content in the control rats in the neurochemical analyses conducted after the T-maze experiment, i.e., after the latency returned to the original level. This experimental result appears to indicate a possible dissociation between the NAc DA content and the latency, and thereby potentially challenges the proposal that tonic DA relates to response vigor (Niv et al., 2007), although the neurochemical analyses may not necessarily reflect tonic DA during task engagement.

### Mechanisms of TD-RPE calculation

PPN contains both neurons representing obtained reward and those representing expected values (Okada et al., 2009), and the former or both have been proposed to contribute to TD-RPE calculation (Kawato and Samejima, 2007; Okada et al., 2009; Cohen et al., 2012; Morita et al., 2012). In reference to these proposals, we assumed that the post-DA-depletion increase of PPN neural activity causes an increase of the gain of one or more terms of TD-RPE, although whether the increase of neural activity indeed indicates a gain increase requires validation (discussed below). We found that the behavioral results of DA depletion could be reproduced when the gain of the obtained-reward-term prominently increased whereas the expected-value-terms entailed no or mild gain increase (Figs. 8 and 9). This is in line with a possibility that DA neuron-projecting PPN neurons contribute the obtained-reward term, but scarcely the expected-value-terms, to TD-RPE. DA neurons receive excitatory inputs also from other regions, including the laterodorsal tegmental nucleus (LDT), lateral hypothalamus, and subthalamic nucleus (STN), that are suggested to convey reward/reinforcement information (Dautan et al., 2016; Tian et al., 2016; Xiao et al., 2016). PPN in our model could additionally/alternatively represent these nuclei. Among them, STN neurons were shown to exhibit a transient decrease of the firing rate and a persistent increase of burst firing after DA depletion (Ni et al., 2001) while STN lesion reversed the increase in PPN firing rate (Breit et al., 2001), but their overall impacts on DA neurons remain to be fully elucidated.

There have been proposals that direct projections from striatum to DA neurons contribute to RPE calculation (e.g., Wörgötter and Porr, 2005). Although optogenetic activation of the direct projections evoked weak or no inhibition (Chuhma et al., 2011; Xia et al., 2011; Bocklisch et al., 2013), the direct inputs from NAc to ventral tegmental area (VTA) DA neurons were recently shown to preferentially activate slow metabotropic GABA_B_ receptors (Edwards et al., 2017). It then seems possible that these inputs could contribute to the previous-value term of RPE, as previously proposed (Houk et al., 1995), or alternatively, generation of the previously reported ∼4-Hz oscillation (Fujisawa and Buzsáki, 2011), which could implement “time steps.” On the other hand, DA neurons have been suggested to receive GABA_A_ inputs from the nearby substantia nigra pars reticulata (SNr; Tepper et al., 1995) that are considered to have a prepotent effect compared to the inputs from the striatum or globus pallidus (Tepper and Lee, 2007). Recent work indicated that activation of SN GABAergic neurons negatively impacts reinforcement learning (Ramayya et al., 2017). It has also been demonstrated that VTA GABAergic neurons represent expected reward (Cohen et al., 2012) and their inputs to DA neurons provide the previous-value term of RPE (Eshel et al., 2015). Also, optogenetic stimulation of D1 MSNs in NAc activated VTA DA neurons through inhibition of VTA GABAergic neurons (Bocklisch et al., 2013; Keiflin and Janak, 2015). The CS-TD hypothesis (Morita et al., 2012, 2013; Morita, 2014; Morita and Kawaguchi, 2015), proposing that D1 and D2 MSNs contribute the current and previous-value terms to TD-RPE with opposite signs via SNr (or potentially VTA) GABAergic neurons, appears to be in line with these latter findings.

DA neurons receive direct projections from neurons in various brain regions (Watabe-Uchida et al., 2012). A recent study (Tian et al., 2016) revealed that these neurons, even those within a single region such as the striatum, exhibited a variety of activity patterns and appeared to represent obtained reward, expected reward, or both. The authors argued that this result was at odds with predictions of theoretical models assuming that each brain region just contains neurons representing a particular term of RPE. Our present model, however, predicts that striatal MSNs exhibit a variety of activity patterns (Fig. 15). Neurons receiving impacts (directly or indirectly) from the striatum, including those in the globus pallidus and STN, are also expected to exhibit various activity patterns. In addition, striatal MSNs, as well as many other neurons projecting to DA neurons, are also receivers of DA inputs, and thus their activity could potentially reflect DA neurons’ response to obtained reward even if they primarily represent expected-reward values. Although such effects were not incorporated into our model, features in the results on DA depletion shown in Figs. 8*A* and 14*D* could be largely preserved when additionally assuming mild modulations of MSNs by DA/TD-RPE at the previous time step, more specifically, up/down [down/up]-regulations of D1/D2 MSNs by positive [negative] DA/TD-RPE at the previous time step [see Materials and Methods for details; data not shown, but can been seen in the ModelDB (Fig. S2)]. Nonetheless, properly examining the effects of the reciprocal interactions of MSNs and DA neurons requires more detailed biophysical modeling in the future.

### Possible rationale and mechanisms for the decay of learned values

In our model, we assumed the decay/forgetting of learned values, which played critical roles in the simulations. There have been studies in humans (Erev and Roth, 1998; Dai et al., 2015; Niv et al., 2015) and animals (Ito and Doya, 2009; Khamassi et al., 2015) suggesting that choice behavior could be well fitted by models assuming value decay, although the tasks were different from those modeled in our present study, and the formulas/assumptions for the models and/or value decay were different from our present model. Regarding the time-scales, one of those studies (Niv et al., 2015) reported that the mean best-fit rate of decay (assumed only for the weights of features not included in the chosen option in their model) per trial was ∼0.420∼0.466. In our present study, the rate of decay (assumed for all the values) was assumed to be 0.01 per time step, which corresponds to 0.395 (= 1 − 0.99^50^) per 50 time steps and thus appears to be comparable to or milder than the rate reported in the above study, although direct comparison is difficult because of the differences in the tasks and models. Crucially, if the learned values always decay at a constant rate, the value memory would be almost completely lost while subjects are not engaged in the task even though the rate is small, but this is obviously not adaptive. Therefore, so as to be behaviorally adaptive, decay should occur when and only when subjects are engaged in the relevant task so that the value-storing synapses receive background inputs related to the task. This could potentially be realized through induction of weak synaptic plasticity by the background inputs, in particular, those coming from task-representing cortical activity, although actual physiologic mechanisms need to be explored. Occurrence of decay specifically for the duration of task engagement could also be realized through mechanisms analogous to reactivation-induced memory destabilization, a phenomenon considered to be linked with memory reconsolidation (Lee et al., 2017).

### Limitations and perspectives

Effort-related impairments caused by DA depletion after completion of learning have been thought to relate to DA’s functions other than its role in learning, i.e., TD-RPE coding. Our results suggest that those impairments could still relate to DA’s TD-RPE coding if completion of learning in fact means a dynamic equilibrium where learning and forgetting are balanced. Our results also suggest that behavioral effects of DA receptor antagonisms could reflect changes in DA’s TD-RPE signals given that many neurons expressing DA receptors in turn modulate DA neuronal activity directly or indirectly. Our TD-RPE–centric view was partly motivated by the recently reported ramping/sustained DA signals, which were argued to be a departure from the conventional view that RPE is encoded by phasic DA, while tonic DA has separate functions, although the ramping/sustained DA signals can still be in line with the (phasic)-DA = TD-RPE hypothesis as has been shown (Gershman, 2014; Morita and Kato, 2014).

However, the lack of distinction between tonic and phasic DA signals and the ignorance of DA’s roles other than the TD-RPE coding are still important limitations of the present work. Although tonic DA and phasic DA can be cooperative, as a way of their interaction, gain modulation of phasic signals by tonic activation has been suggested (Grace, 2016). Moreover, tonic DA and phasic DA can be differentially regulated (Floresco et al., 2003), and tonic DA can even be antagonistic to phasic DA in certain conditions, e.g., when DA released presynaptically, independent of cell-body activation, binds to D2Rs on DA axons (Grace, 1991). Also, although DA’s TD-RPE-coding is assumed to be accomplished by DAergic modulation of synaptic plasticity, DAergic modulation of instantaneous neuronal responsiveness would also directly affect behavior. Both types of DAergic modulations were incorporated into the different model mentioned before (Collins and Frank, 2014), while TD-type RPE was not, and future models should explore how all the features can be incorporated at once.

Another important limitation of the present work lies in our assumption on the secondary effects of DA depletion. We assumed an increase of the gain of variables represented by PPN neurons, in reference to the experimentally observed increase in the PPN firing rate. This assumption could be largely valid if the observed firing-rate increase was due to an increase in the gain of input-output relation, i.e., a multiplicative increase of the output, although the increase in the baseline output was not incorporated into the model. However, the observed firing-rate increase could instead reflect an additive, rather than multiplicative, increase in the PPN output. Recent work (Geng et al., 2016) reported that the firing rate of putative cholinergic PPN neurons during locomotion (5.607 ± 0.438 spikes/s) was higher than the rate during rest (2.871 ± 0.264) in control rats, and both rates were higher in rats with DA depletion (10.410 ± 1.455 and 4.092 ± 0.341). The average increases of these firing rates by DA depletion appear to imply a gain modulation, but this point was not tested in that study, and possible biophysical mechanisms remain unclear. Moreover, what occurs in reward-related activity of PPN neurons also remains to be seen. Therefore, at present, gain increase of the obtained-reward-representing PPN input should be regarded as an assumption that needs to be carefully validated.

## Synthesis

Reviewing Editor: Leonard Maler, University of Ottawa Decisions are customarily a result of the Reviewing Editor and the peer reviewers coming together and discussing their recommendations until a consensus is reached. When revisions are invited, a fact-based synthesis statement explaining their decision and outlining what is needed to prepare a revision will be listed below. Note: If this manuscript was transferred from JNeurosci and a decision was made to accept the manuscript without peer review, a brief statement to this effect will instead be what is listed below.

I have carefully read the original JNS reviews, the rebuttal to those reviews and the second reviews and rebuttals.

I agree that the comments by Reviewer #1 suggested that he/she had simply not read the Ms with sufficient care.

In any case, all the pertinent points raised by this reviewer were fully addressed.

Reviewer #2 raised many sensible and helpful points. The authors have taken the time and effort to use the critique to enhance their Ms.

Reviewer #2 is fully satisfied.

The model presented in this Ms is quite complicated and, although I do a lot of modelling, I did not have time to fully comprehend the fine points of the analyses in this Ms (I don't work in this field).

However, I was able to check on every point raised by all the reviewers and to assess how relevant this Ms was for the field.

I conclude that the model analysis presented in this paper will be an important contribution to the now extensive theoretical literature on reinforcement learning.
