# Supplementary figures and images for: A Neural Circuit Mechanism for the Involvements of Dopamine in Effort-Related Choices: Decay of Learned Values, Secondary Effects of Depletion, and Calculation of Temporal Difference Error
Source: eNeuro. 2018 Feb 21;5(1):ENEURO.0021-18.2018. doi: 10.1523/ENEURO.0021-18.2018 (PMC5820541; doi:10.1523/ENEURO.0021-18.2018)

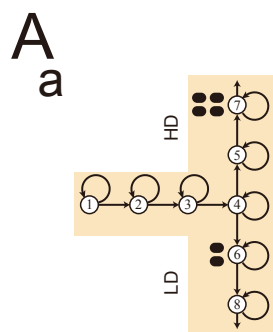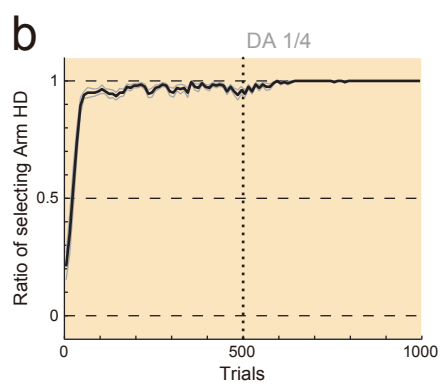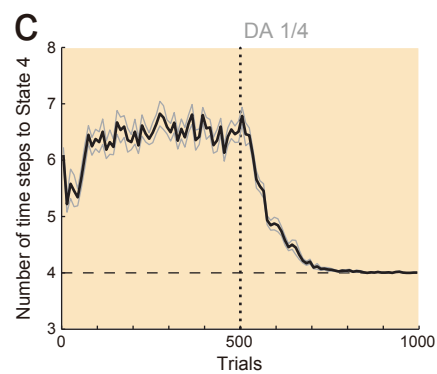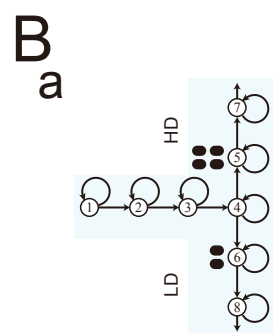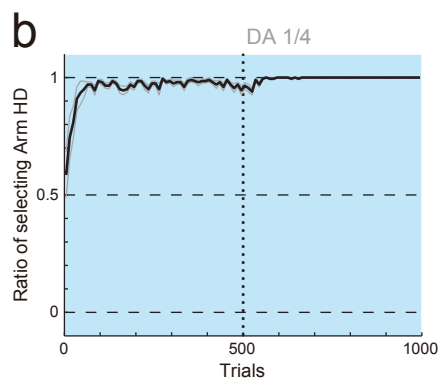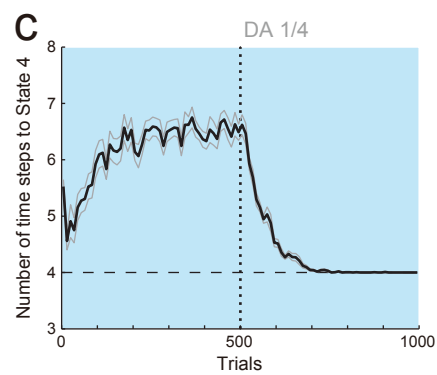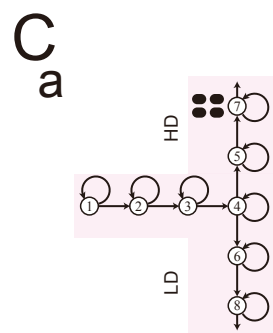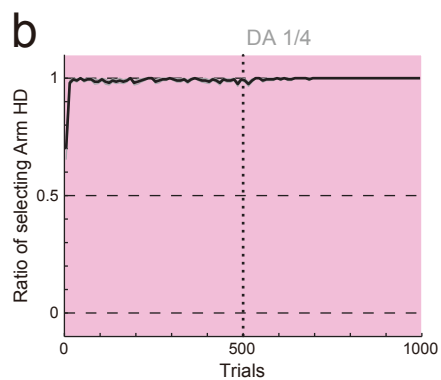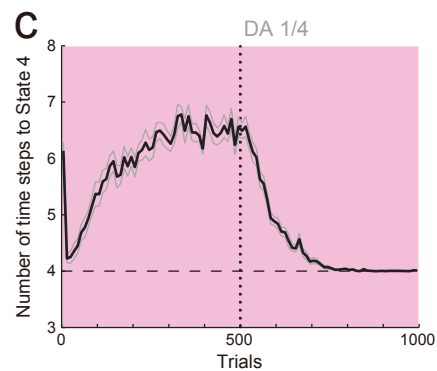

**Figure S1**

Supplement: Extended Data 1 — Computer code: makefigures.m: running this file (script M-file) will reproduce all the figure panels showing simulation results in the article, as well as two supplementary figures (which are also attached here) simTmaze.m: function M-file for simulating the effort-related T-maze task, which is called in makefigures.m ana1.m–ana7.m: function M-files for analysis and plotting, which are called in makefigures.m rantwi.mat: saved data (more specifically, outputs of rand(‘twister’)), which are used in makefigures.m to create exactly the same figures as presented in the article mean2.m, std2.m, sem2.m: function M-files to calculate mean, std, and sem for input numbers omitting NaN Fig. S1.pdf, Fig. S2.pdf: supplementary figures created by makefigures.m How to run the makefigures.m files: (1) Running makefigures.m will reproduce exactly the same results shown in the article (i.e., using the same sets of MATLAB-built-in pseudo-random numbers). (2) Running makefigures.m with “samerand = 1” (line 4) changed to “samerand = 0” will result in conducting all the simulations with new sets of Matlab built-in pseudorandom numbers. (3) Running makefigures.m with “mod_negativeTDE = 1” (line 19) changed to “mod_negativeTDE = 0” will result in conducting simulations assuming size reduction of TD-RPE–dependent value update by DA depletion with the assumption that the size reduction is applied only when TD-RPE is nonnegative. Download Extended Data 1, ZIP file. [file sup_enu-eN-NWR-0021-18-s02.zip › FigS1.pdf]

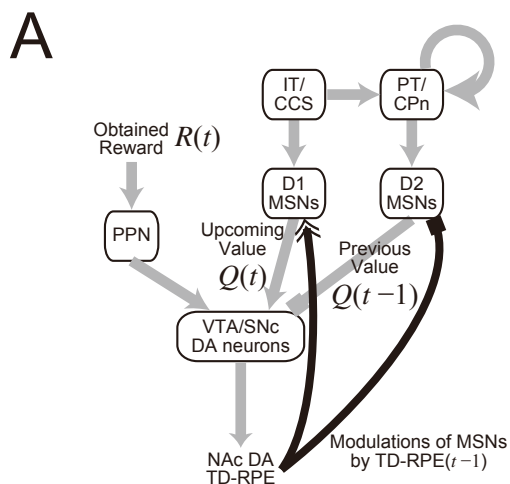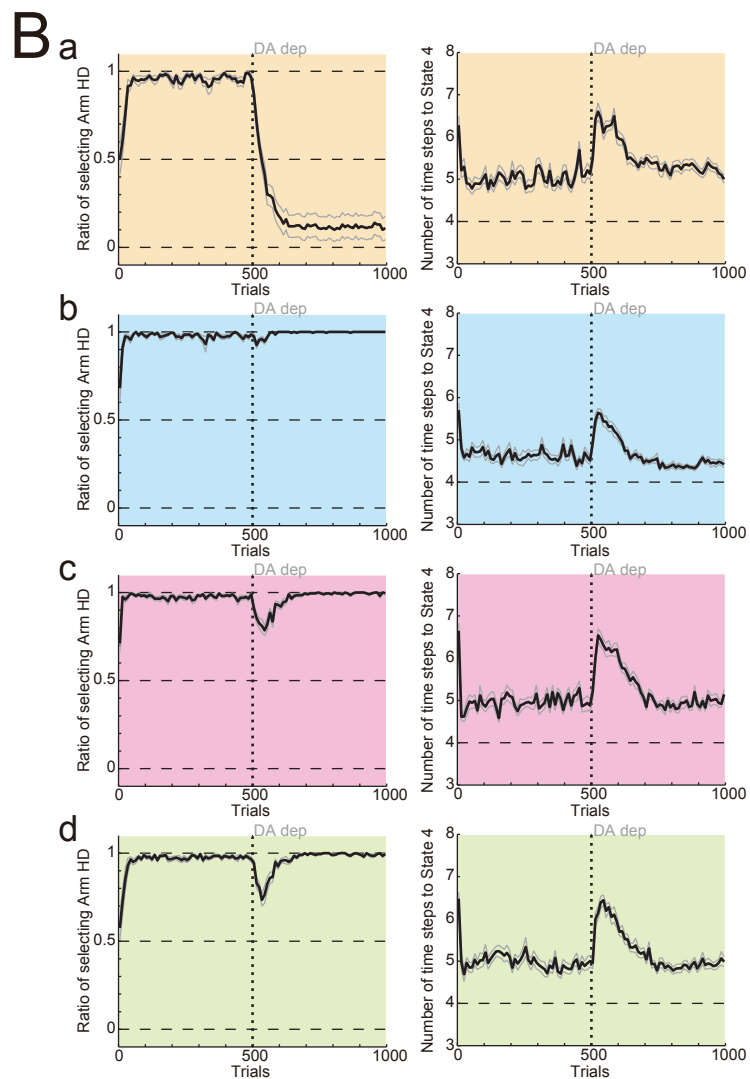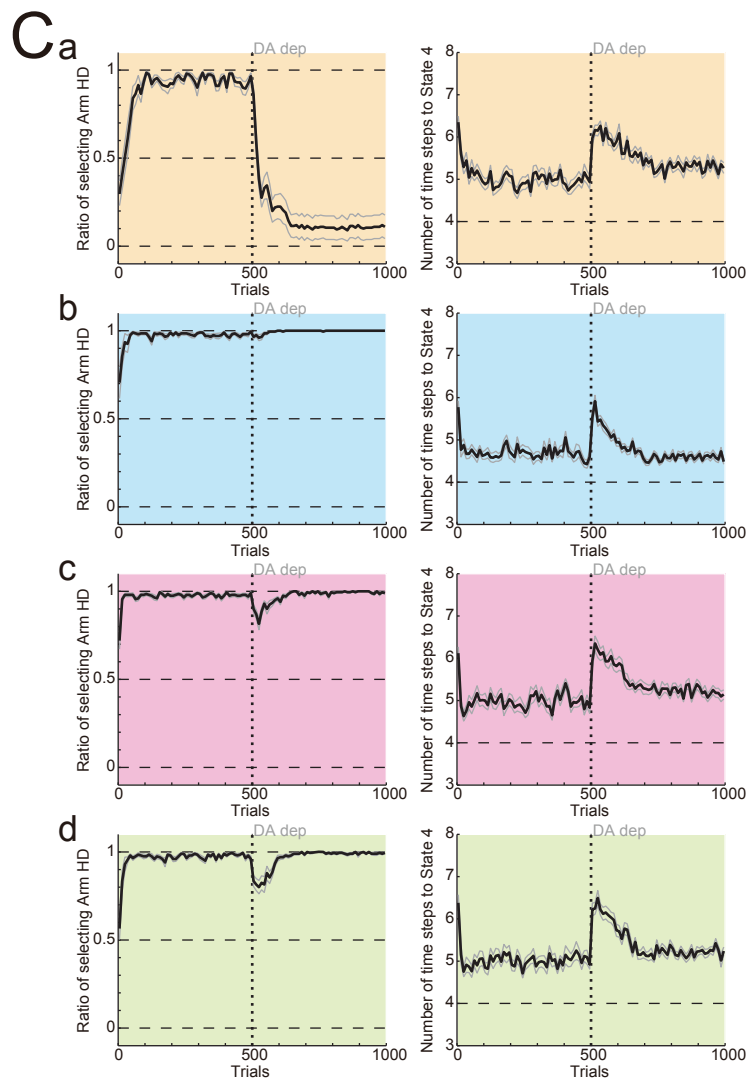

**Figure S2**

Supplement: Extended Data 1 — Computer code: makefigures.m: running this file (script M-file) will reproduce all the figure panels showing simulation results in the article, as well as two supplementary figures (which are also attached here) simTmaze.m: function M-file for simulating the effort-related T-maze task, which is called in makefigures.m ana1.m–ana7.m: function M-files for analysis and plotting, which are called in makefigures.m rantwi.mat: saved data (more specifically, outputs of rand(‘twister’)), which are used in makefigures.m to create exactly the same figures as presented in the article mean2.m, std2.m, sem2.m: function M-files to calculate mean, std, and sem for input numbers omitting NaN Fig. S1.pdf, Fig. S2.pdf: supplementary figures created by makefigures.m How to run the makefigures.m files: (1) Running makefigures.m will reproduce exactly the same results shown in the article (i.e., using the same sets of MATLAB-built-in pseudo-random numbers). (2) Running makefigures.m with “samerand = 1” (line 4) changed to “samerand = 0” will result in conducting all the simulations with new sets of Matlab built-in pseudorandom numbers. (3) Running makefigures.m with “mod_negativeTDE = 1” (line 19) changed to “mod_negativeTDE = 0” will result in conducting simulations assuming size reduction of TD-RPE–dependent value update by DA depletion with the assumption that the size reduction is applied only when TD-RPE is nonnegative. Download Extended Data 1, ZIP file. [file sup_enu-eN-NWR-0021-18-s02.zip › FigS2.pdf]
